# Epigenetic Regulation in Cardiovascular Diseases: Mechanisms and Therapeutic Targets

**DOI:** 10.1002/mco2.70774

**Published:** 2026-05-28

**Authors:** Wangzheqi Zhang, Chenyang Mu, Rui Zhao, Runwei Ma, Xuehai Liu, Haoling Zhang, Zengwu Wang, Jing‐jing Zhang

**Affiliations:** ^1^ School of Anesthesiology Naval Medical University Shanghai China; ^2^ Department of Nursing Ren Ji Hospital, Shanghai Jiao Tong University School of Medicine Shanghai China; ^3^ Gansu University of Chinese Medicine Lanzhou Gansu Province China; ^4^ Fuwai Yunnan Hospital, Chinese Academy of Medical Sciences, Affiliated Cardiovascular Hospital of Kunming Medical University Kunming China; ^5^ Yunnan Provincial Cardiovascular Clinical Medical Center Kunming China; ^6^ Yunnan Provincial Cardiovascular Clinical Medical Research Center Kunming China; ^7^ Clinical College of Traditional Chinese Medicine Gansu University of Chinese Medicine Lanzhou Gansu Province China; ^8^ Department of Biomedical Sciences, Advanced Medical and Dental Institute Universiti Sains Malaysia Penang Malaysia; ^9^ Division of Prevention and Community Health, National Center for Cardiovascular Disease, National Clinical Research Center of Cardiovascular Disease, State Key Laboratory of Cardiovascular Disease Fuwai Hospital, Peking Union Medical College & Chinese Academy of Medical Sciences Beijing China

**Keywords:** cardiovascular diseases, chromatin remodeling, enhancer rewiring, epigenetic regulation, noncoding RNAs, precision epigenetic therapy

## Abstract

Cardiovascular diseases (CVDs) arise as the product of multiple factors, including genetic predisposition and environmental exposure, but the molecular and cellular interplays underlying such a pathogenetic process leading to sustained pathological conditions remain largely unknown. A growing body of evidence indicates that epigenetic mechanisms, including DNA methylation, histone modifications, chromatin remodeling, noncoding RNAs, and RNA modifications, are important for cardiovascular development, adaptation, and disease. However, findings from these studies are fragmented across different genomic contexts or disease‐focused scenarios, lacking systematic integration for a global view connecting epigenetic dynamics to cardiovascular phenotypes and their clinical implications. An overall picture of epigenetic modulation in CVDs is therefore provided. Common epigenetic mechanisms are described in a cell type and disease stage‐specific context, and the roles of environmental and lifestyle factors in remodeling the cardiovascular epigenome are illustrated. Novel epigenetic biomarkers and therapeutic interventions are also assessed. Emphasis is placed on epigenomic plasticity, enhancer focused control, and network‐level reprogramming as key concepts that drive disease remodeling. By unifying mechanistic understanding with translational evidence, this review frames CVD as a disorder of stabilized regulatory states and defines avenues for precision epigenomic manipulation.

## Graphical abstract

Environmental and lifestyle exposures reshape the cardiovascular epigenome through coordinated regulation of DNA methylation, histone modifications, noncoding RNAs, and RNA methylation (m^6^A). These multilayered mechanisms drive enhancer‐centered regulatory network remodeling and epigenomic plasticity in cardiovascular diseases, highlighting emerging opportunities for epigenetic biomarkers and precision‐targeted therapies.

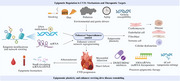

## 1 Introduction

Cardiovascular diseases (CVDs) remain the leading cause of morbidity and mortality worldwide despite substantial advances in prevention, diagnosis, and treatment. Major clinical entities—including atherosclerosis, hypertension, myocardial infarction (MI), heart failure (HF), and arrhythmias—continue to impose a significant global health burden. Although therapeutic strategies such as lipid‐lowering interventions and blood pressure control have improved outcomes in selected populations, the overall incidence of CVDs continues to rise due to aging populations, metabolic disorders (e.g., obesity), and persistent exposure to adverse environmental and lifestyle factors [1].

Over the past decades, genetic studies—particularly genome‐wide association studies—have identified numerous loci associated with CVD risk and have provided insights into pathways involving lipid metabolism, inflammation, vascular remodeling, and cardiac structure. However, genetic variation alone explains only a limited proportion of disease heritability. Most identified variants are located in noncoding regions and have modest predictive value at the individual level. Moreover, purely genetic models cannot adequately account for the substantial influence of environmental exposure, lifestyle behaviors, and life‐course factors on cardiovascular outcomes [2, 3].

This gap has led to growing interest in regulatory mechanisms that bridge genetic susceptibility and environmental influence. Epigenetic regulation—defined as heritable changes in gene expression without alterations in DNA sequence—provides such an interface. Key epigenetic mechanisms, including DNA methylation, histone modifications, chromatin remodeling, noncoding RNAs (ncRNAs), and RNA methylation, dynamically regulate transcriptional programs in response to developmental cues, metabolic states, mechanical stress, and inflammatory signals. Unlike static genetic variation, epigenetic modifications are dynamic and context‐dependent, enabling cells to integrate external stimuli into relatively stable regulatory states [4, 5].

In the cardiovascular system, epigenetic regulation plays a critical role in maintaining the balance between cellular stability and adaptive plasticity. Cardiomyocytes, endothelial cells (ECs), vascular smooth muscle cells (VSMCs), fibroblasts, and immune cells share the same genome but exhibit distinct functional phenotypes due to cell‐type‐specific epigenomic landscapes. Disruption of this balance between stability and flexibility has emerged as a hallmark of CVD pathogenesis. Advances in high‐resolution epigenomic profiling, single‐cell sequencing, and multiomics integration have further revealed that disease‐associated epigenetic alterations are frequently concentrated at regulatory elements such as enhancers and superenhancers, leading to large‐scale rewiring of gene‐regulatory networks rather than isolated gene changes [5, 6].

In addition, epigenetic regulation provides a mechanistic link between environmental and lifestyle factors and long‐term cardiovascular health. Factors such as diet, physical activity, smoking, air pollution, and psychosocial stress can induce persistent epigenetic changes that may contribute to disease susceptibility and progression, supporting the concept of epigenetic memory. Meanwhile, the reversible nature of certain epigenetic modifications offers opportunities for therapeutic intervention, although challenges remain regarding specificity, delivery, and long‐term safety [4, 7].

This review provides a comprehensive overview of epigenetic regulation in CVDs. The major epigenetic mechanisms underlying cardiovascular pathophysiology are summarized, followed by a discussion of how environmental and lifestyle factors shape the cardiovascular epigenome. Emerging epigenetic biomarkers for diagnosis and prognosis are examined, and current and potential epigenetic therapeutic strategies are evaluated. Key challenges and future directions are highlighted, with emphasis on epigenomic plasticity and network‐level regulation as central concepts in understanding and treating CVDs.

## 2 Major Epigenetic Mechanisms Underlying Cardiovascular Diseases

Epigenetic control involves several intertwined molecular layers which determine gene expression without changing the sequence of the encoded deoxyribonucleic acid (DNA). In CVDs, such layers are more akin to a regulatory stack, as opposed to parallel “modules”: upstream cues (such as genetic background, hemodynamic load, hypoxia, inflammation, and metabolic stress) get interpreted into cell‐state‐specific transcriptional programs that can outlive the initiating signal [8, 9]. Of note, the same stimulus almost never evokes a homogeneous epigenetic response throughout the heart and vasculature; rather, it is processed through lineage history, chromatin context, and ongoing signaling that likely underlie why related risk exposures can lead to different phenotypes (e.g., atherosclerosis vs. adverse remodeling) [2, 4].

A salient feature of epigenetic regulation is its ability to make stability compatible with plasticity. Some of these marks support long‐term maintenance of cell identity, while others allow rapid and partially reversible adaptation to severe transient stress (ischemia–reperfusion, cytokine exposure, and mechanical stretch). If this balance is shifted—especially during chronic stress—cells may get “stuck” in maladaptive programs driving hypertrophy, fibrosis, endothelial dysfunction, or inflammatory activation [2, 4]. Yet causality is not one‐to‐one: the epigenetic signatures of many diseases likely contain both drivers and passengers of tissue remodeling, which cannot be disentangled without thoughtful study design and cell‐resolved profiling [5].

Cell‐type specificity is thus not a detail but a central organizing principle. Cardiomyocytes, ECs, VSMCs, fibroblasts, and immune cells have the same genome but distinct epigenomic landscapes that influence differential (albeit shared) systemic signals [2, 3]. This view has been bolstered by recent single‐nucleus and multiomic analysis of human cardiomyopathy, which suggested that disease‐associated regulatory change tends to localize to cellular states rather than being uniformly distributed across bulk tissue [10]. Concurrently, spatial multiomics approaches are beginning to reveal that epigenetic–transcriptional programs are also spatially localized within the injured myocardium and vasculature (e.g., border zones postinfarction), suggesting that “where” a cell may have substantial impact on its regulatory program [11].

Technological progress has also changed focus from promoter‐centric storylines to enhancer and 3D‐genome–aware models. Genome‐wide profiling of the epigenome has revealed that disease‐associated changes are frequently enriched at enhancers/superenhancers, coordinated with differential chromatin accessibility, transcription factor occupancy, and long‐range interactions [5, 6]. More broadly, these observations promote a less linear perspective in which epigenetic mechanisms rewire regulatory circuits by shifting their dynamics rather than just toggling genes on and off again.

Following are the subsections that elaborate on some of the central epigenetic layers involved in CVD. We begin with DNA methylation, which is established as a largely stable but remodelable regulatory landscape, and progress to histone modification, and chromatin remodeling, processes that can introduce complexity and dynamics into the enhancer activity and global chromatin context. We then move on to ncRNAs, which serve as epigenetic modifiers, and finally we describe RNA methylation as a fast‐responding layer capable of redirecting stress responses by cooperation with the chromatin‐associated regulators.

### 2.1 DNA Methylation Modifications

**FIGURE 1:**
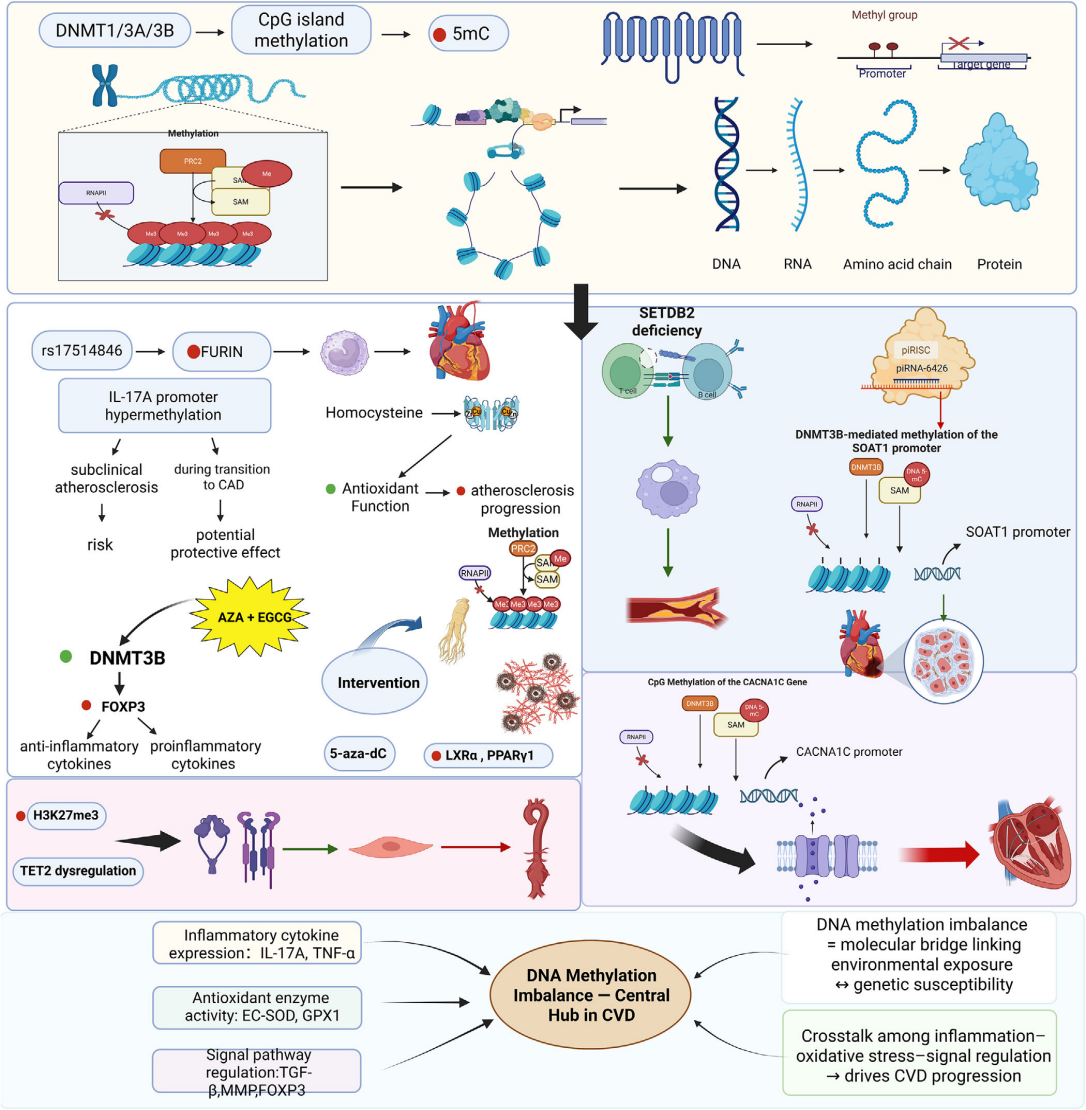
DNA methylation and its role in CVDs. CpG island methylation is catalyzed by DNA methyltransferases (DNMT1, DNMT3A, and DNMT3B), resulting in gene silencing. This diagram shows how DNA methylation mediates the effects of inflammation factors (IL‐17A and SOAT1) involved in atherosclerosis and other CVDs. The therapeutic potential of the DNMT inhibitors, such as 5‐Aza and epigallocatechin gallate (EGCG), is also discussed.

DNA methylation is among the most extensively studied epigenetic mechanisms in cardiovascular biology and disease. It predominantly occurs at cytosine residues within cytosine–phosphate–guanine (CpG) dinucleotides and is deposited by DNA methyltransferases, while active and passive demethylation pathways enable remodeling across timescales. In cardiovascular cells, DNA methylation can operate as a comparatively stable—yet not immutable—constraint on transcription, integrating developmental history with environmental and pathological cues to shape longer term gene expression programs [8, 12]. That said, the functional consequences of methylation changes are rarely uniform: depending on genomic context (promoter vs. enhancer), baseline chromatin state, and transcription factor availability, methylation may correlate with repression, permissiveness, or in some cases reflect downstream chromatin remodeling rather than primary regulation [5]. In this context, DNA methylation functions not only as a transcriptional constraint but also as a mediator linking inflammatory signaling to long‐term cardiovascular phenotypes. A schematic overview of these mechanisms is provided in Figure 1.

#### 2.1.1 Cell‐Type‐Specific Methylomes and Interpretability Constraints

A general feature of CVD is that methylation landscapes are highly cell‐type specific. Specific methylomes are set up in cardiomyocytes, ECs, VSMCs, fibroblasts, and immune cells to enable differences in lineage commitment, metabolic need, and stress experience [2, 3]. This cell specificity is not only descriptive: it dictates how each of these populations interprets inflammatory signals, metabolic dysregulation, and mechanical forces to modulate both propensity and course. Practically, it also creates a recurring interpretability constraint—bulk‐tissue methylation profiles may conflate genuine regulation with shifts in cellular composition during inflammation or fibrosis, which can inflate false mechanistic confidence if not accounted for [5].

#### 2.1.2 Vascular Disease: Endothelial Dysfunction and VSMC Phenotypic Switching

In the vascular compartment, aberrant DNA methylation is frequently associated with endothelial dysfunction and atherogenesis. Altered methylation at promoters and enhancers of genes involved in inflammation, oxidative stress, and leukocyte adhesion may support a more persistent endothelial activation state, thereby facilitating monocyte recruitment and plaque progression [13, 14, 15]. Nevertheless, the directionality can be context‐sensitive; for some loci, methylation differences may represent a record of exposure (e.g., smoking‐related epigenetic memory) rather than a direct driver of lesion growth [4, 7]. Where functional experiments exist, they suggest that methylation‐associated regulatory changes can influence endothelial inflammatory tone, which plausibly compounds other risk pathways rather than acting alone.

In VSMCs, methylation is implicated in phenotypic switching from a contractile to synthetic state—an axis relevant to neointima formation and vascular remodeling. Disease‐associated methylation patterns may interact with transcription factor networks and chromatin accessibility to enable proliferative and migratory programs, including extracellular matrix deposition [16, 17]. Importantly, VSMC switching is often heterogeneous within lesions; therefore, “average” methylation signatures may underestimate state‐specific regulatory extremes that matter biologically.

#### 2.1.3 Myocardial Remodeling: Hypertrophy, Metabolic Reprogramming, and Progression to Failure

In the myocardium, DNA methylation changes have been linked to pathological hypertrophy, metabolic reprogramming, and transition toward HF. Disease‐associated methylation alterations are reported in genes governing contractile function, calcium handling, stress signaling, and energy metabolism, with the cumulative effect of reshaping transcriptional networks that drive maladaptive remodeling [18, 19]. Notably, rather than isolated promoter events, many studies increasingly point to coordinated changes across regulatory regions—consistent with a model in which methylation participates in network‐level reprogramming. However, caution remains warranted: without temporal resolution and cell‐specific perturbation, it can be difficult to exclude the possibility that some methylation signatures arise secondarily to cardiomyocyte stress, immune infiltration, or fibroblast expansion [5].

#### 2.1.4 Environmental “Memory” and Persistence of Cardiovascular Risk

DNA methylation provides a plausible mechanistic bridge between exposure history and long‐term cardiovascular risk. Diet, smoking, psychosocial stress, and environmental pollutants have each been associated with persistent methylation changes at cardiovascular‐relevant loci in epidemiological and experimental contexts [4, 7]. In some settings, methylation differences remain detectable after exposure cessation, supporting an “epigenetic memory” concept. Yet persistence does not automatically imply causality; methylation may also function as a biomarker of cumulative exposure. Dissecting these possibilities likely requires longitudinal sampling, causal inference frameworks, and integration with chromatin accessibility/transcriptional output.

#### 2.1.5 Translational Implications and What Still Limits the Field

Several challenges still complicate interpretation of disease‐associated methylation changes. Many datasets are cross‐sectional and rely on bulk tissue, limiting causal inference and obscuring cell‐specific effects. Moreover, methylation drivers may differ across disease stages; early lesions may show more reversible regulatory plasticity, whereas chronic disease may involve more entrenched epigenomic states. Emerging single‐cell and integrative multiomics approaches address these limitations by linking methylation changes to transcription and phenotype with greater resolution [5]. Collectively, current evidence supports DNA methylation as a foundational CVD mechanism—particularly as a stability layer that records and constrains stress‐adaptive programs—while highlighting the need for stage‐ and cell‐resolved designs to distinguish drivers from correlates.

### 2.2 Histone Modifications

Histone modifications represent a dynamic epigenetic control layer that allows for rapid and context‐dependent gene regulation in cardiovascular cells. Covalent post‐translational modifications of histone tails, such as acetylation, methylation, phosphorylation, and ubiquitination, affect nucleosome stability and regulate the recruitment of regulatory complexes in a manner that determines chromatin availability and transcription competency [8, 12]. Histone marks change more temporally than DNA methylation, which may be suitable for encoding acute stimuli such as stress during ischemia (and in aging muscle), inflammatory activation, and mechanical overload.

In general, histone acetylation is associated with gene transcription activation by neutralizing the interaction between histones and DNA, as well as increasing open chromatin. In CVD, dysregulated acetylation leads to persistent activation of inflammatory, hypertrophic, and fibrotic gene programs. Enhanced protein acetylation at regulatory elements of proinflammatory genes in endothelial and immune cells could promote vascular inflammation, leukocyte trafficking, and the development of atherosclerosis [20, 21]. In cardiomyocytes, acetylation‐based transcriptional reprogramming may be relevant for both metabolic adaptation and hypertrophy, therefore being involved in the transition from compensatory/remodeling to failure during chronic stress [22, 23]. But acetylation changes are not evenly distributed: disease‐associated modifications converge at enhancers rather than promoters in a manner more consistent with an enhancer amplification model, where locally concentrated chromatin alterations result in widespread network‐wide transcriptional impacts [4, 24, 25].

In contrast, histone methylation has context‐specific functions based on the residue and degree of methylation, as well as chromatin location. Repressive methylation marks contribute to establishing stable silencing of developmental and lineage‐inappropriate genes, breaking such alliances has also been associated with fetal gene re‐expression in hypertrophy as well as HF, supporting the notion that erosion of adult transcriptional identity in part explains the phenotypic maladaptation [26, 27, 28]. On the other hand, activating methylation marks may promote expression of genes promoting angiogenesis, mitochondrial integrity, and contractility. Crucially, disease‐linked changes in histone methylation tend to occur in a coordinated fashion throughout regulatory regions, reflecting a model where methylation is involved in network‐level reorganization of gene regulation rather than switching individual genes [4, 24, 25].

One persistent issue—applicable to both proposed mechanisms and clinical translation—is cell‐type selectivity. The same histone‐modifying enzyme in cardiomyocytes, ECs, VSMCs, and fibroblasts may have opposing effects because chromatin context and transcription factor landscape vary between lineages [2, 4]. Such context dependency may in part explain why broad perturbation of the histone‐modifying pathways sometimes results in a mixed phenotypic outcome, and why selective targeting at precise stages or cells appears to be increasingly required for translational success.

Temporal dynamics also matter. One potential explanation is that early adaptive responses involve reversible changes in acetylation that maintain a level of transcriptional flexibility, and this is followed by more stable reprogramming associated with chronic disease through methylation that locks the cells into maladaptive states [5]. This stage‐specific transition holds practical implications: interventions that seem efficacious in early remodeling could attenuate or even rebound when the epigenomic state reaches its steady state.

**FIGURE 2:**
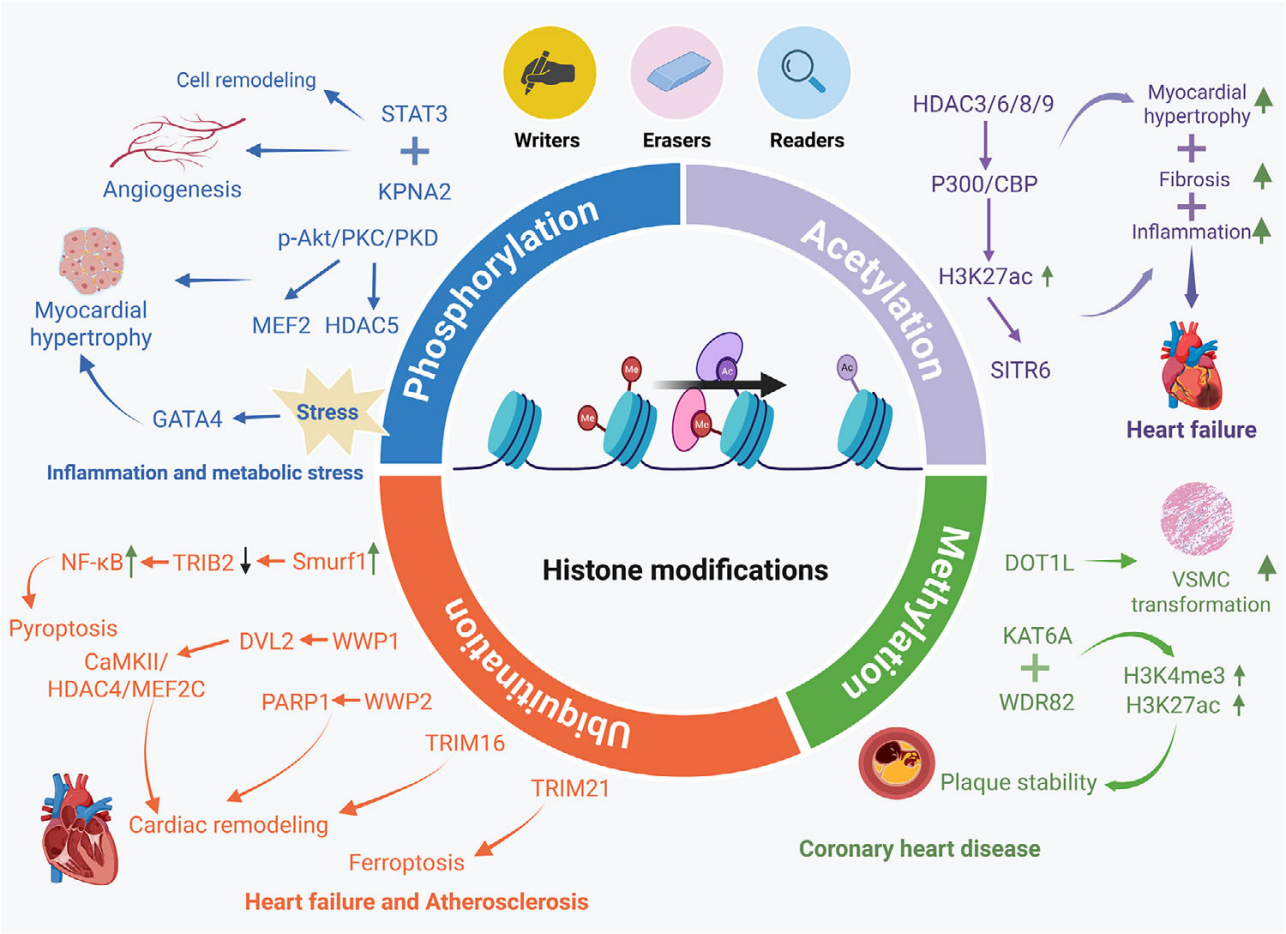
Histone modifications in cardiovascular pathology. Histone acetylation, methylation, phosphorylation, and ubiquitination are important for the control of chromatin structure and transcription. HDACs, HATs, and EZH2 act as key regulators of heart disease including hypertrophy, fibrosis, and HF. NF‐κB, MAPK, TGF‐β signaling pathways converge on these changes to drive disease development.

Some of the most relevant major histone modification‐dependent regulatory pathways involved in CVD are depicted in Figure 2.

### 2.3 Chromatin Remodeling

The positioning of nucleosomes, the accessibility of the chromatin and its higher order genome organization are controlled by adenosine triphosphate (ATP)‐dependent chromatin remodeling. Through direct modulation of chromatin architecture, remodeling complexes control the accessibility to regulatory DNA and thereby tune the transcriptional capacity in a mark‐independent manner [8, 12]. In the context of CVD, chromatin remodeling is emerging as a central mechanism for driving stress‐responsive gene regulation, especially by altering enhancer repertoires.

Disease‐triggered remodeling can further serve to open up access at stress‐responsive enhancers, while limiting activation of elements associated with developmental programs, and hence magnify transcriptional networks which some cell types have been previously shown to associate with inflammation, fibrosis, and hypertrophy in a cell‐type‐specific manner [4, 24, 25]. One interpretation of this is that chromatin accessibility alterations can happen before any observable steady‐state transcriptional changes, acting as an early checkpoint rather than strictly reading out downstream gene expression [5]. However, to demonstrate “upstream causality,” profiling studies must ideally include time‐resolved data in the context of perturbations—otherwise, accessibility changes might still be reactive.

In addition to local access, chromatin remodeling also contributes to the 3D genome organization. Changes in chromatin looping and topologically associating domain (TAD) structure may shift enhancers with respect to target promoters, leading to abnormal long‐range contacts that sustain the disease gene program within the myocardium and vasculature [5, 6]. This spatial dimension also helps explain why other disease‐associated variants and epigenetic changes map to distal regulatory elements: they can alter enhancer–promoter contacts even if promoter marks are unaffected.

The chromatin is also regulated by DNA methylation, histone marks, and RNA‐mediated regulation. Accessibility changes may impact deposition and interpretation of histone marks, and DNA methylation could reinforce remodeling barriers at specific sites. In truth, such layers form hierarchical regulatory cascades with initial recruitment‐mediated chromatin opening followed by subsequent recruitment of transcription factors and chromatin writers/readers that are reinforcing a disease‐associated regulatory state [4, 5]. This nested perspective is attractive for embodiment because it offers many points of potential intervention—but also a warning that targeting any one layer might not be enough if stabilizing mechanisms in parallel are left intact.

**FIGURE 3:**
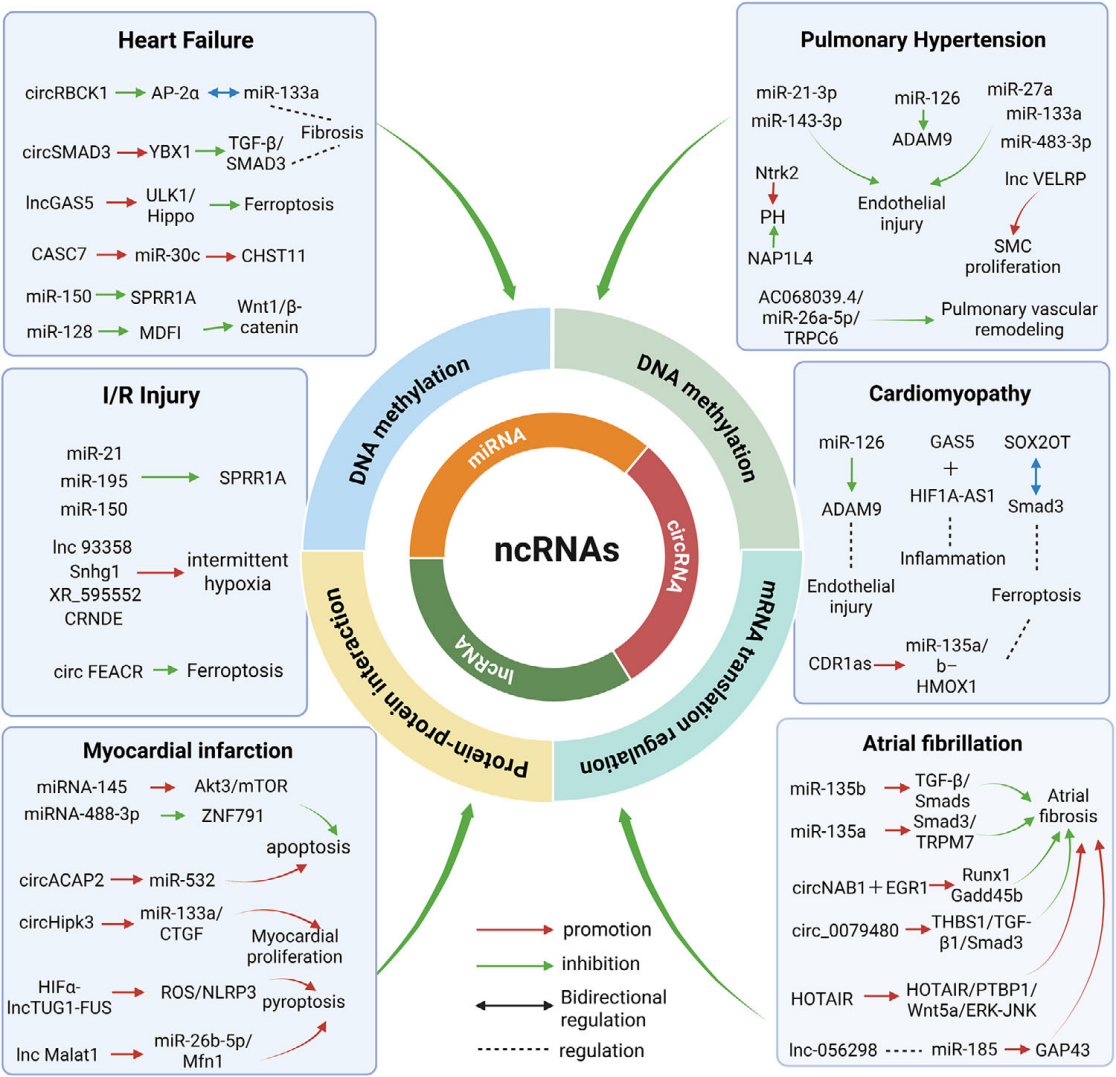
The role of ATP‐dependent chromatin remodeling in CVDs. ATP‐dependent chromatin remodeling complexes: CHD7, EZH2, and the SWI/SNF complex are important regulators of chromatin condensation and histone methylation. These epigenetic changes control the silencing and reactivating of fetal genes, which results in cardiac fibrosis, hypertrophy, and arrhythmias. The schematic describes how chromatin relaxation is combined with transcriptional regulation in CVD.

Prominent chromatin remodeling policies and their functions in enhancer rewiring and 3D genome reorganization are summarized in Figure 3.

### 2.4 ncRNAs With Epigenetic Regulatory Functions

ncRNAs form a very diverse group of transcripts with various functions. In cardiovascular research, ncRNAs are frequently discussed in a general fashion; only some of the ncRNAs have direct and convincing epigenetic relevance—that is, active or inactive interaction with chromatin, epigenetic modifiers, or transcriptional regulatory complexes. It is important to keep this distinction because the post‐transcriptional regulation should not be confused with epigenetic control and for clarity regarding mechanism [29, 30].

MicroRNAs (miRNAs) are mainly post‐transcriptional regulators; however, a subset may affect epigenetic landscapes indirectly via regulation of DNA methylation or histone‐modifying enzymes. Through such interactions, miRNAs can have an epigenetic effect at the systems level: by changing the levels of chromatin regulators and modulating transcriptional state [31, 32, 33]. Indeed, such effects also tend to be context‐dependent—manifesting most prominently in the setting of chronic stress—and as such must be viewed in the framework of a specified cell type and disease stage rather than indiscriminately extrapolated across CVD [4, 5].

Long noncoding RNAs (lncRNAs) afford the most robust evidence for direct epigenetic contributions to CVD. A majority of lncRNAs are nuclear, where they serve as scaffolds, guides, or decoys for chromatin‐modifying enzymes and transcription factors. This allows for locus‐specific addition or removal of epigenetic marks and contributes to programs associated with hypertrophy, fibrosis, angiogenesis, and inflammation [34, 35, 36, 37]. A fundamental aspect is the cell‐type specificity: most lncRNAs are expressed in a limited number of cells and act only in specific settings (e.g., cardiomyocytes upon pressure overload, fibroblasts during fibrotic remodeling) [2, 4]. This specificity can engender large phenotypic changes despite modest alterations in expression, but also poses translational hurdles related to trafficking and off‐target effects.

Circular RNAs (circRNAs) have been largely considered as miRNA sponges, but a fraction of these molecules might be involved in epigenetic control: either by interacting with the transcriptional machinery or even exerting their action modulating the activity of epigenetic‐modifying proteins. The available information is premature and patchy at best, and in most cases the effect of many circRNAs on chromatin could only be inferred but not actually demonstrated [38, 39]. Where evidence is found, it is becoming ever more desirable to combine transcriptomics with chromatin assays, and perturbations in order to pass beyond the level of correlation [5].

**FIGURE 4:**
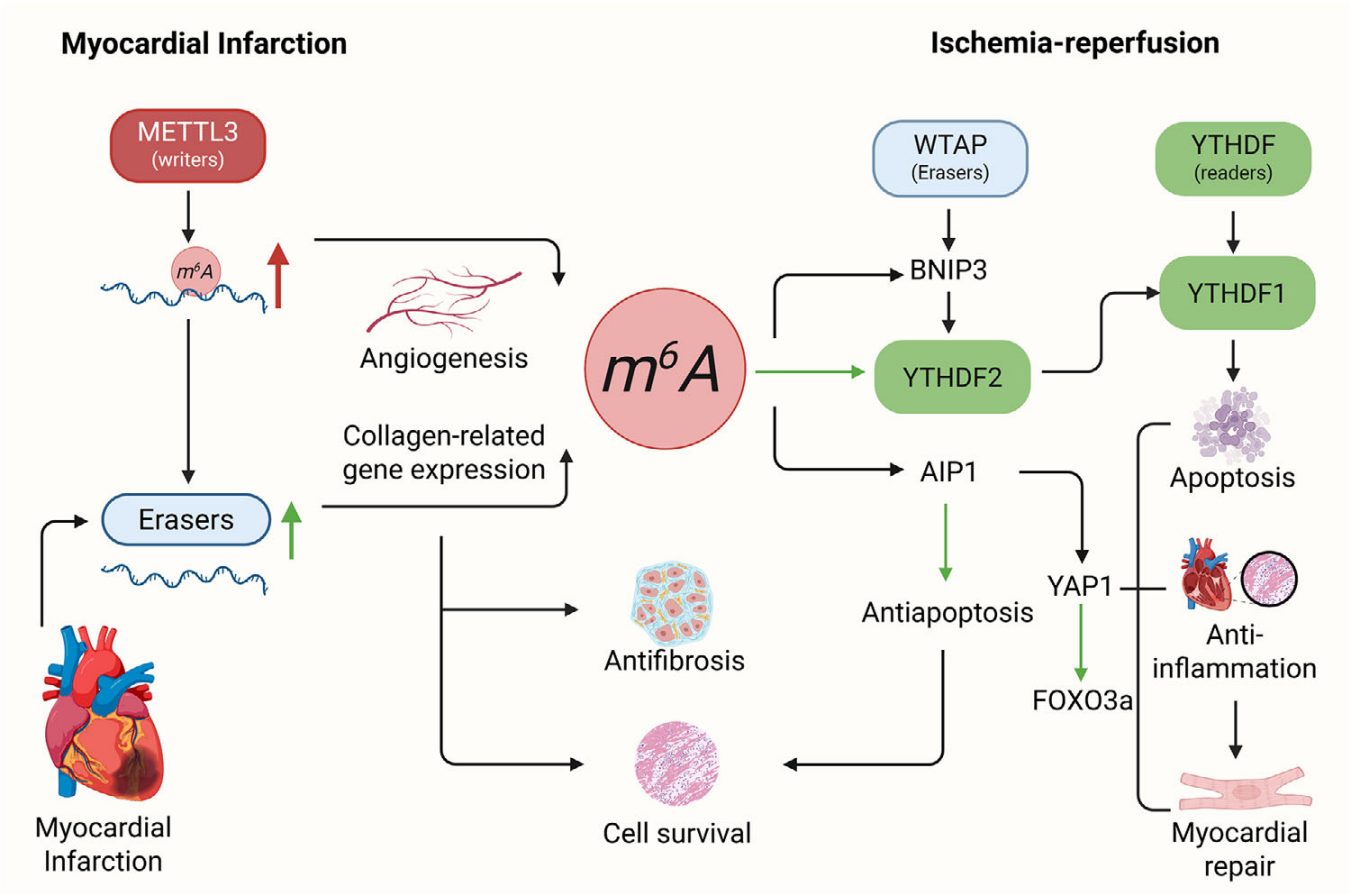
Regulatory roles of ncRNAs in CVDs. ncRNAs, including miRNAs, lncRNAs, and circRNAs, are crucial in the control of cardiovascular diseases such as HF, MI, pulmonary hypertension (PH), and cardiomyopathies. These ncRNAs target DNA methylation, protein function, and gene expression to regulate inflammation, fibrosis, and metabolism. Molecules such as miR‐33, MALAT1, and circMAP3K5 can be observationally used as key molecules in fibrotic remodeling and metabolic regulation.

An overview of the major pathways by which ncRNA epigenetic modifiers act upon chromatin and regulatory complexes in CVD is presented in Figure 4. In Table 1, an extensive summary of ncRNAs with known or putative epigenetic roles, their molecular targets, and related signaling pathways is presented.

**TABLE 1:** Major molecular mechanisms of ncRNAs regulation in CVDs.

ncRNA type (miRNA/lncRNA/circRNA)	Specific name	Target gene/binding target	Involved pathway or signaling mechanism	Functional effect (pathogenic/protective)	CVD type	References
miRNA	miR‐1	HCN2, HCN4 (hyperpolarization‐activated cyclic nucleotide‐gated channels); binds to the 3′‐UTR of HAX1 to suppress its expression	Regulates apoptosis and inflammation via the HCN2/HCN4axis; inhibits the HAX1/HMG20A/Smads axis and TGF‐β1 signaling	Protective (suppresses apoptosis, enhances cell viability and anti‐inflammatory activity; inhibits inflammation and myocardial fibrosis)	CHF; MI	[40, 41]
	miR‐34a	Sirt1, cyclin D1, and Bcl2	ADAR2/miR‐34a regulatory axis; lncRNA Kcnq1ot1/miR‐34a‐5p/Sirt1 signaling pathway	Pathogenic (impairs antiapoptotic and reparative mechanisms, amplifying injury effects)	MI; arsenic trioxide‐induced cardiotoxicity	[42, 43]
	miR‐21	CPT1A; SPRY1	Targets CPT1A to regulate macrophage polarization and mitochondrial autophagy; miR‐21/SPRY1/ERK/mTOR signaling pathway	Pathogenic (upregulation aggravates myocardial injury and fibrosis; promotes the development and progression of diabetic cardiomyopathy)	CHF; diabetic cardiomyopathy	[44, 45]
	miR‐126	PIK3R2; RGS3	PI3K/AKT signaling pathway; inhibition of RGS3 signaling	Protective (upregulation suppresses cardiac hypertrophy); pathogenic (induces cardiomyocyte ion channel dysfunction)	Cardiac hypertrophy; takotsubo cardiomyopathy	[46, 47]
	miR‐155	SHP2; IL‐6R	ERK1/2 pathway and NLRP3 inflammasome‐associated pathway; IL‐6R/JAK2/STAT3 signaling pathway	Pathogenic (overexpression exacerbates myocardial necroptosis and apoptosis; suppresses cardiomyocyte proliferation, aggravating cardiac injury)	Myocardial ischemia; MI	[48, 49]
	miR‐29	SERPINH1; KCNJ12, KCNIP2	Regulates fibrosis‐related processes by inhibiting SERPINH1; modulates potassium channel genes to influence arrhythmogenesis	Protective (reduces aging‐associated tissue fibrosis); pathogenic (upregulation increases the risk of ischemic arrhythmias)	Myocardial fibrosis; ischemic arrhythmias	[50, 51]
lncRNA	MALAT1	miR‐26b‐5p; Mfn1;miR‐20b‐5p; beclin1	miR‐26b‐5p/Mfn1 axis regulates mitochondrial dynamics and apoptosis; miR‐20b‐5p/beclin1 axis regulates autophagy	Protective (preserves cardiac microvascular function); pathogenic (promotes cardiomyocyte injury)	MI; myocardial ischemia	[52, 53]
	H19	ERS apoptosis‐related proteins (PERK/IRE1/CHOP, etc.); miR‐let‐7g and TGFβR1; E2F transcription factor 1‐mediated EZH2	Activates the PI3K/AKT/mTOR signaling pathway; H19/let‐7g/TGFβR1 axis mediates endothelial‐to‐mesenchymal transition; E2F1/EZH2 pathway regulates right ventricular remodeling	Protective (preserves myocardium, inhibits apoptosis and fibrosis); pathogenic (promotes hypoxia‐induced PAH; drives right ventricular decompensation in PAH)	Diabetic cardiomyopathy; hypoxia‐induced PAH; right ventricular failure in PAH	[54, 55, 56]
	MEG3	miR‐129‐5p; ATG14/Akt; miR‐181a‐5p; ABCA1; miR‐223; TRAF6	miR‐129‐5p/ATG14/Akt axis regulates apoptosis and autophagy; miR‐181a‐5p/ABCA1 axis; miR‐223/TRAF6/NF‐κB signaling axis	Pathogenic (promotes cardiomyocyte apoptosis and autophagy; accelerates progression of viral myocarditis); protective (enhances cholesterol efflux)	HF; CAD; viral myocarditis	[57, 58, 59]
	TUG1	HIF‐1α, VEGF‐α; miR‐34a‐5p	Exosomal lncRNA TUG1/HIF‐1α/VEGF‐α axis; induces autophagy via regulation of miR‐34a‐5p	Pathogenic (suppresses angiogenesis; exacerbates myocardial ischemia/reperfusion injury)	MI; myocardial ischemia	[60, 61]
	HOTAIR	FUS, SIRT3; PTBP1, Wnt5a; miR‐17‐5p, STAT3	Inhibits cardiomyocyte pyroptosis via the FUS/SIRT3 axis; Wnt/ERK/JNK signaling pathway; HOTAIR/miR‐17‐5p/STAT3 axis suppresses apoptosis	Protective (inhibits high glucose–induced cardiomyocyte pyroptosis; protects against myocardial ischemia/reperfusion injury); pathogenic (promotes atrial fibrosis)	Diabetic cardiomyopathy; AF; myocardial ischemia/reperfusion injury	[62, 63, 64]
	XIST	miR‐217;miR‐340‐5p	XIST/miR‐217 axis regulates oxidative stress and apoptosis; XIST/miR‐340‐5p/CCND1 axis regulates apoptosis and stress response	Pathogenic (promotes oxidative injury and apoptosis; aggravates myocardial ischemia/reperfusion injury)	Myocardial ischemia/reperfusion injury	[65, 66]
circRNA	CDR1as	miR‐135a; miR‐135b; miR‐7a	miR‐135a/HMOX1 and miR‐135b/HMOX1 signaling axes; Cdr1as/miR‐7a/PARP‐SP1 signaling axis	Pathogenic (modulates the onset and progression of CHF; promotes cardiomyocyte apoptosis and enlarges infarct size)	CHF; MI	[67, 68]
	HIPK3	HuR, β‐TrCP; miR‐17‐3p, ADCY6; miR‐33a‐5p, IRS1; Notch1 acetylation and miR‐133a/CTGF	circHIPK3/HuR/β‐TrCP/p21 axis regulates cellular senescence; circHIPK3/miR‐17‐3p/ADCY6axis regulates calcium signaling; circHIPK3/miR‐33a‐5p/IRS1axis; stabilizes Notch1 via N1ICD acetylation and sponges miR‐133a to upregulate CTGF	Protective (inhibits cardiomyocyte senescence, improves cardiac function; suppresses cardiomyocyte apoptosis; promotes cardiogenesis and angiogenesis, protects against cardiac fibrosis); pathogenic (promotes HF and post‐MI fibrosis)	Aging‐related cardiac dysfunction; HF; MI	[69, 70, 71, 72]
	Foxo3	Cytokines IL‐10, IL‐33, IL‐34; BTG2 and AMPK‐FOXO3a pathway‐related molecules; VEGF‐A, VEGF‐B	Activation of Foxo3 via the PP2A/p‐Akt pathway; AMPK‐FOXO3a signaling pathway; PI3K/AKT‐FoxO3a‐VEGF‐A/B signaling cascade	Protective (immunoregulatory protection that promotes cardiac repair; mitigates cardiotoxicity; optimizes energy metabolism and cardiac function)	MI; doxorubicin‐induced cardiotoxicity; HF	[73, 74, 75]
	PVT1	miR‐30a‐5p; miR‐125b‐5p	circPVT1/miR‐30a‐5p/miR‐125b‐5p axis regulates cardiac fibrosis	Pathogenic (promotes cardiac fibrosis)	Ischemic HF	[76]
	ITCH	Transcription factor P73; miR‐330‐5p and its target genes SIRT6/BIRC5/ATP2A2	Tax1bp1/ITCH/P73/BNIP3 apoptosis pathway; circITCH/miR‐330‐5p/SIRT6‐survivin‐SERCA2a axis	Pathogenic (promotes cardiomyocyte apoptosis, exacerbates HF); Protective (protects myocardium and alleviates doxorubicin‐induced cardiotoxicity)	HF; doxorubicin‐induced cardiotoxicity	[77, 78]
	β‐catenin	FOSL1, FOSL2 (AP‐1 transcription factor subunits); E‐cadherin; glycolytic enzymes (HK2/PFK/PKM2/LDH)	Wnt‐β‐catenin‐FOSL signaling pathway promotes fibrosis/hypertrophy; regulation of β‐catenin degradation via GSK‐3β/APC; β‐catenin‐glycolysis axis promotes macrophage inflammation	Pathogenic (promotes right ventricular remodeling and failure; accelerates atrial fibrosis and AF progression; promotes PH development)	PAH right HF; AF	[79, 80, 81]

### 2.5 RNA Methylation

RNA methylation has become a prevalent regulatory level which is increasingly relevant to cardiovascular biology. Of > 100 reported RNA modifications, N6‐methyladenosine (m^6^A) is not only the most abundant one but also the best studied. Although the effects of RNA methylation are post‐transcriptional, influencing RNA stability, splicing, export, translation, and decay, its activity may contribute to long‐term changes in cellular programming under chronic stress and can be considered functionally adjacent to classical epigenetic modifications [82, 83, 84]. A useful analogy to consider the role of RNA methylation in CVD is that it behaves like a rapid‐response rheostat: it can potentially dial up or down both the magnitude and duration of stress‐adaptive gene expression, and by doing so indirectly affect the state of chromatin and transcription at large, through modulating levels of chromatin and transcriptional regulators [4, 85].

A notable feature is the capacity for stress‐responsive behavior. m^6^A profiles can also change in cardiomyocytes, ECs, and VSMCs under ischemia, pressure overload, oxidative stress, and metabolic dysfunction, influencing transcripts including those that are involved in energy metabolism, calcium handling, stress signaling, and inflammation [86, 87, 88]. However, responses are frequently dependent on cell type and context: the same stressor can elicit divergent m^6^A signatures across lineages, implying that RNA methylation is more likely to adjust state transitions than implement a uniform disease program [5].

Recent mechanistic studies have started to supply more concrete points of reference for translational consideration. For instance, decreased FTO expression in postinfarction conditions has been shown to be associated with increased fibrotic response and intervention experiments suggest that the FTO‐mediated m^6^A regulation can impact collagen synthesis through specific downstream targets [89]. In ischemia/hypoxia models, m^6^A modification (by the methyltransferase‐like 3 [METTL3] enzyme) was suggested to regulate cardiomyocyte injury through autophagy signaling by directly targeting ATG7 messenger RNA (mRNA) for degradation and reader‐mediated decay as a potential mechanistic link between stress sensing and cell fate decision [90]. While these help to strengthen the case for biological plausibility, generalization remains uncertain, as effects may depend on model system, stage of disease, and the profile of writers/erasers/readers in each cell type.

And RNA methylation is not alone. Through its modulation of chromatin regulators, signaling intermediates, and transcription factor transcripts’ stability and translation, m^6^A can modulate the supply of proteins that act directly on chromatin, thus indirectly impacting epigenomic plasticity [4, 85]. This cross‐talk may, in part, explain why RNA methylation is increasingly discussed in the context of CVD alongside the chromatin machinery—despite having a primary substrate of RNA instead of DNA or histones.

Interpretation is, however, still limited by a number of constraints despite improvement. A great many studies are based on correlative mapping or global perturbation of the m^6^A machinery, which can potentially induce profound secondary effects. Technical caveats associated with site‐specific readout and connecting individual modification events to phenotype add an additional layer of complexity to inference. As a result, existing data suggest that RNA methylation is at most a context‐specific regulator (e.g., under ischemic injury or in activated fibroblasts), as opposed to an all‐encompassing master switch [5].

Despite mounting interest, the function of RNA methylation in CVD remains ill‐defined. Most analyses are correlative in nature or involve global perturbation of the RNA methylation machinery; thus, we cannot infer causality. In addition, it is technically challenging to map site‐specific RNA modifications and to connect these modifications with functional correlates, which hampers mechanistic inference. Therefore, currently available data suggest a contributory rather than primary role of RNA methylation in cardiovascular epigenetic control [5].

The limitations mentioned above are now starting to be challenged by new methods combining single‐cell resolution, temporal profiling, and targeted perturbation. They are needed to differentiate direct regulatory roles of RNA methylation from the secondary consequences on cellular stress and disease contexts in which modulation of RNA methylation could serve as a therapeutic or biomarker target.

RNA methylation cannot function alone but rather cross‐talks with DNA methylation, histone modification, chromatin reconfiguration, and ncRNA‐affected control. Through regulation of transcript fate and translation, RNA methylation modulates the availability and activity of central regulatory proteins in higher order gene‐regulatory networks. This integrative function places RNA methylation as a fluid amplifier or repressor of epigenetic states, rather than an upstream determinant of cellular identity.

**FIGURE 5:**
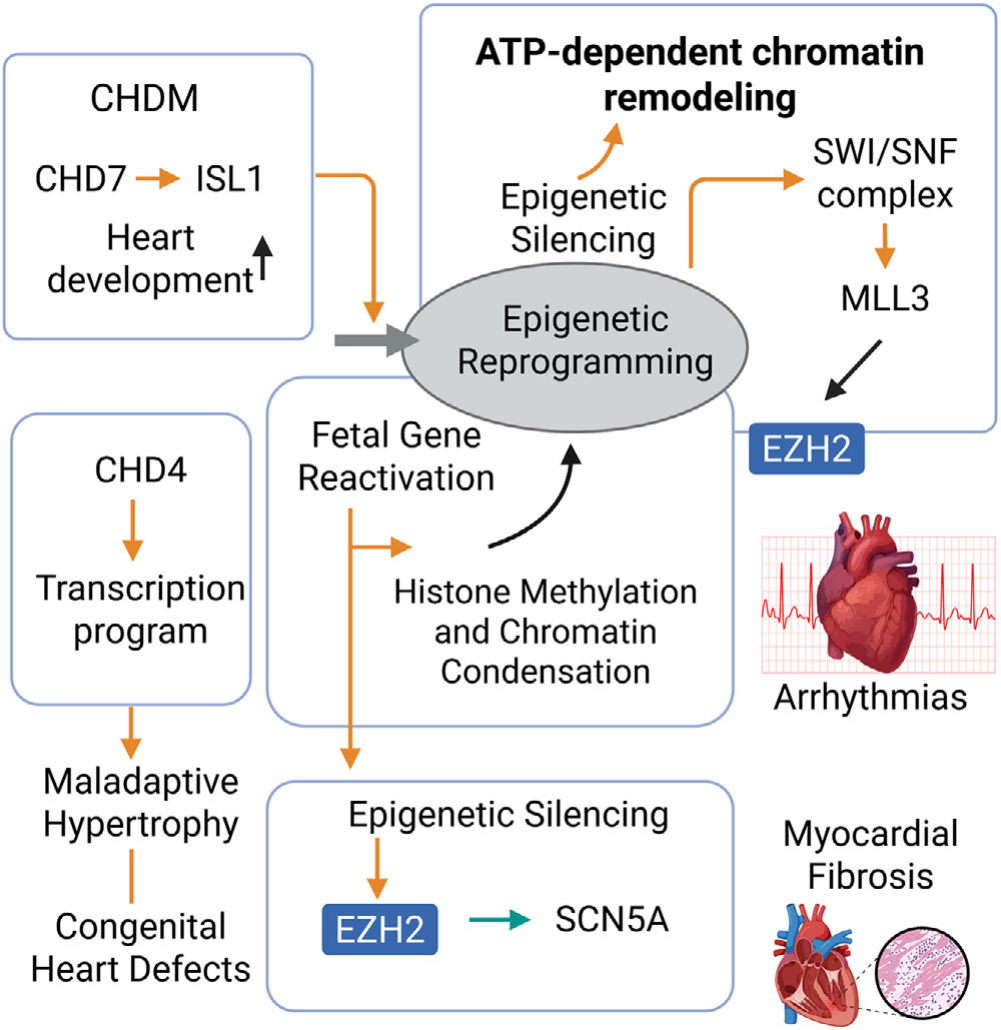
The role of m^6^A modification in CVDs. The m^6^A modification, which is mediated by METTL3, plays a crucial role in myocardial ischemia–reperfusion injury and MI. m^6^A writers, erasers, and readers are involved in angiogenesis, collagen expression, apoptosis, and myocardial repair. m^6^A‐regulated pathways involve the targeting of YTHDF2, AIP1, BNIP3, YAP1, and FOXO3a during inflammation modulation, cell survival, and tissue repair in CVD. The diagram highlights the role of m^6^A‐dependent pathways involving YTHDF2, AIP1, BNIP3, and YAP1, as well as FOXO3a, in regulating inflammatory responses associated with CVD.

Key pathways connecting RNA methylation and stress‐responsive gene regulation as well as the dynamics of the epigenetic network in CVD are depicted in Figure 5. Other more in‐depth studies focused on the role of RNA methylation in CVD models, and stress conditions are outlined in Table 2, thereby achieving a balance between representing extensive literature articles met by the main text narrative style.

**TABLE 2:** Association between epigenetic regulation and common CVDs.

CVD type	Epigenetic mechanism categories: DNA methylation/miRNA/histone modifications, and so forth	Key molecules/biomarkers	Target molecules or regulatory pathways	Functional effects	Clinical significance (diagnosis/prognosis/treatment)	References
HF	RNA methylation	Methyltransferase METTL4, mtDNA 6 mA; piRNA‐6426, DNMT3B, SOAT1	mtDNA promoter, p53‐METTL4 pathway; SOAT1 promoter methylation	Excessive 6 mA leads to mitochondrial dysfunction and HF; inhibits cardiomyocyte oxidative stress/inflammation, alleviates HF	Therapeutic: Inhibition of METTL4 as a novel strategy for HF treatment; targeting piRNA‐6426 as a new direction for HF therapy	[14, 91]
	lncRNA	lncRNA MIAT, SERCA2a, RyR2; GASL1, TGF‐β1 ; GAS5, YAP/TAZ	Regulation of calcium cycling genes (SERCA2a/RyR2) expression; GASL1 inactivates TGF‐β1	Alleviates cardiomyocyte apoptosis, fibrosis, and enhances contractile function; inhibits ferroptosis in cardiomyocytes and improves heart function	Therapeutic: Targeting Miat as a novel strategy for treating cardiac hypertrophy; BMSC‐Exos carrying GAS5 for treating heart failure. Prognostic: GASL1 as a prognostic biomarker and potential therapeutic target	[92, 93, 94]
	miRNA	miR‐222; miR‐103‐3p; HLF; FYCO1	Targeting p53 apoptotic factors, Hmbox1, NFAT3, and other genes; miR‐103‐3p targets HLF	Inhibits pathological remodeling, delays HF; promotes cardiomyocyte apoptosis, inhibits autophagy	Therapeutic: miR‐222 as a potential target for HF treatment; miR‐103‐3p as a potential therapeutic target for HF	[95, 96]
	circRNA	AP‐2α, circRBCK1, miR‐133a; circPALLD, GATA4, PALLD	AP‐2α/circRBCK1 pathway inhibits miR‐133a; GATA4 regulates PALLD transcription	Improves cardiac diastolic dysfunction; involved in the onset and progression of heart failure	Therapeutic: Statins can target this pathway for HFpEF treatment; Diagnostic: Potential diagnostic biomarker and therapeutic target	[37, 97]
PAH	DNA methylation	SIN3a, BMPR2	SIN3a‐DNMT1/EZH2/TET1‐BMPR2 pathway	Inhibits pulmonary artery SMC proliferation and vascular remodeling	Therapeutic: SIN3a gene therapy as a potential treatment strategy for PAH	[98, 99]
	lncRNA	GAS5 and miR‐382‐3p; lncRNA VELRP, WDR5	GAS5 targets miR‐382‐3p pathway; VELRP‐WDR5‐CDK signaling pathway	Inhibits SMC proliferation and migration, promotes autophagy and inhibits angiogenesis; promotes pulmonary artery SMC proliferation and vascular remodeling	Therapeutic: Provides new targets for CTEPH treatment; targeting VELRP as a new direction for PH treatment	[100, 101]
	miRNA	miR‐126; ADAM9; miR‐30d; MTDH; PDE5A; NRF1	miR‐126 targets ADAM9 to regulate endothelial cell survival	Promotes endothelial cell apoptosis and microvascular remodeling	Prognostic: Potential prognostic marker for the onset and progression of COPD‐PH	[87, 102]
	circRNA	circMyst4; circCDR1as; circSIRT1	DDX5/GPX4 pathway, Eef1a1/ACSL4 complex, ferroptosis; CDR1as/miR‐7‐5p axis targets CAMK2D/CNN3; circSIRT1competes with miR‐145‐5p to mediate Akt3 expression	Alleviates pulmonary artery smooth muscle cell (PASMC) ferroptosis, inhibits the progression of hypoxic PH; promotes osteogenic differentiation and calcification of PASMCs; inhibits PASMC proliferation and migration, promotes autophagy and apoptosis, improves PH	Therapeutic: Potential therapeutic target for hypoxic PH; potential therapeutic target for PH vascular calcification	[103, 104, 105]
CAD	lncRNA	TONSL‐AS1; miR‐197; BCL2; LINC01480; AL359237.1	TONSL‐AS1 sponges miR‐197 to regulate BCL2; regulates macrophage M2 infiltration and cardiovascular risk pathways	Inhibits endothelial cell apoptosis and migration; plays a protective role in the progression of ischemic cardiomyopathy	Prognostic: Predicts survival rate in CAD patients; Therapeutic: LINC01480 as a biomarker and therapeutic target for CAD	[106, 107]
	miRNA	miR‐223 and PDGFRβ; miR‐6721‐5p, meta‐VCL	miR‐223 downregulates PDGFRβ to promote VSMC differentiation; miR‐6721‐5p targets the 3′‐UTR of meta‐VCL	Promotes VSMC differentiation and alleviates vascular damage; miR‐6721‐5p upregulation leads to meta‐VCL downregulation, reducing anti‐inflammatory factors	Diagnostic: Identifying high‐risk patients, potential therapeutic target; miR‐6721‐5p as a diagnostic biomarker for CAD	[108, 109]
	circRNA	circARCN1; circZBTB46	Regulates HuR‐USP31‐NF‐κB pathway; binds with hnRNPA2B1 to modulate PTEN/AKT/mTOR pathway	Promotes macrophage accumulation and inflammation, exacerbates AS; promotes cell proliferation and migration, inhibits apoptosis, drives AS	Therapeutic: circARCN1 as a potential target for AS‐associated CAD; Diagnostic: CAD diagnostic biomarker and potential therapeutic target	[110, 111]
MI	circRNA	circACAP2 and miR‐532; circHipk3	Promotes miR‐532 maturation to induce cardiomyocyte apoptosis; Notch1 pathway, miR‐133a/CTGF axis	Promotes cardiomyocyte apoptosis leading to MI; promotes cardiomyocyte proliferation and angiogenesis	Diagnostic: circACAP2 and miR‐532 as potential biomarkers for MI diagnosis; Therapeutic: Targets for treating HF after MI	[72, 112]
	miRNA	miR‐145; miR‐488‐3p	Akt3/mTOR signaling pathway‐related autophagy; miR‐488‐3p targets and inhibits ZNF791	Inhibits myocardial cell apoptosis induced by MI; alleviates myocardial cell apoptosis induced by AMI	Therapeutic: Regulation of autophagy and antiapoptosis as a potential therapeutic target	[113, 114]
	lncRNA	HIF‐1α; lncRNA‐TUG1; FUS; lncRNA Malat1	HIF‐1α/TUG1/FUS axis → ROS/NLRP3 pyroptosis pathway; miR‐26b‐5p/Mfn1pathway	Promotes mitochondrial damage and cardiomyocyte pyroptosis; promotes microvascular repair, inhibits mitochondrial apoptosis	Therapeutic: TUG1 silencing inhibits myocardial injury; provides a therapeutic target for microcirculation repair after MI	[52, 115]
AF	lncRNA	LncRNA HOTAIR;Wnt5a; PTBP1; lncRNA‐056298; cfa‐miR‐185; GAP43	HOTAIR/PTBP1/Wnt5a/ERK‐JNK signaling pathway; lncRNA‐056298 sponges miR‐185 to upregulate GAP43	Promotes atrial fibrosis and drives AF progression; promotes cardiac autonomic remodeling, leading to AF susceptibility	Therapeutic: Potential antifibrosis treatment target; provides new targets for the recurrence mechanism after AF radiofrequency ablation	[63, 116]
	circRNA	circNAB1, NAB1‐356protein; circ_0079480, miR‐338‐3p, THBS1	Binds with EGR1 to regulate Runx1 and Gadd45b transcription; THBS1/TGF‐β1/Smad3 signaling pathway	Inhibits atrial fibrosis, inflammation, and AF susceptibility; promotes atrial fibroblast proliferation, migration, and fibrosis	Therapeutic: Provides potential strategies and targets for AF treatment; offers potential therapeutic targets for AF‐associated atrial fibrosis	[117, 118]
	miRNA	miR‐135b, TGF‐β/Smads pathway molecules; miR‐135a, Smad3, TRPM7	TGF‐β/Smads signaling pathway; Smad3/TRPM7 pathway	Inhibits atrial fibrosis, alleviates AF; regulates atrial fibrosis involved in the onset of AF	Therapeutic: Quercetin as a potential drug for AF treatment; miR‐135a as a potential therapeutic target for AF	[119, 120]
Cardiomyopathy	miRNA	miR‐130b‐3p; GPX4; ACSL4; miR‐133a; COL1A1	ACSL4, PRKAA1, AMPK/mTOR signaling pathway; miR‐133a/COL1A1, inflammatory mediators	Inhibits ferroptosis, improves cardiac function and tissue damage; improves cardiac function and alleviates myocardial fibrosis	Therapeutic: Provides novel treatment targets for septic cardiomyopathy; intravenous injection of hUC‐MSCs for DCM therapy	[121, 122]
	lncRNA	Lnc DCRT; lncCHKB‐DT; FUS; ALDH2	DCRT/PTBP1/NDUFS2 pathway; CHKB‐DT binding FUS → stabilizing ALDH2 mRNA	Maintains mitochondrial complex I function and antioxidant activity; sustains mitochondrial energy metabolism and ATP production	Therapeutic: Coenzyme Q10 may partially treat DCM; novel gene therapy target for DCM	[123, 124]
	circRNA	circHIPK3; miR‐29b‐3p; Col1a1; Col3a1; circHIPK3; PTEN	circHIPK3 sponges miR‐29b‐3p → upregulates collagen expression; circHIPK3/PTEN pathway	Promotes myocardial fibrosis and impairs cardiac function; circHIPK3 downregulates PTEN to inhibit cardiomyocyte apoptosis	Therapeutic: Silencing circHIPK3 attenuates fibrosis; potential cardioprotective target	[125, 126]

## 3 Environmental and Lifestyle Modulation of the Cardiovascular Epigenome

Environmental and lifestyle factors are major determinants of CVD risk and progression. While traditional epidemiological studies have long established associations between diet, physical activity, smoking, psychosocial stress, environmental pollution, and cardiovascular outcomes, epigenetic regulation provides a mechanistic framework through which these exposures may be biologically embedded. By modulating DNA methylation, histone modifications, chromatin accessibility, and RNA‐based regulatory processes, environmental cues can reshape cardiovascular epigenomic landscapes in a context‐ and cell‐type–dependent manner, thereby influencing long‐term gene‐regulatory states rather than transient transcriptional responses alone [4, 7].

Temporal variability over the life course is a key feature of environmentally induced epigenetic regulation. Acute exposures tend to induce reversible epigenetic changes that mediate adaptation over short‐term timescales, whereas chronic or repeated (and perhaps developmental) exposure may result in the stabilization of maladaptive epigenetic states that remain in place after cessation of exposure. This idea of exposure‐induced epigenetic trajectories has gained further support from long‐term human studies and highlights a mechanistic underpinning for how early‐life exposure to transient environmental insults can convey long‐lasting cardiovascular risk [5, 127].

### 3.1 Diet, Metabolic Status, and Physical Activity

Dietary composition and metabolic status exert profound influences on the cardiovascular epigenome. Nutrient availability affects substrate‐dependent epigenetic modifications—including DNA methylation and histone acetylation—by altering intracellular pools of methyl donors, acetyl coenzyme A (acetyl‐CoA), and other metabolic intermediates. Through these mechanisms, metabolic–epigenetic coupling regulates transcriptional programs involved in lipid handling, mitochondrial function, and inflammatory signaling, thereby shaping susceptibility to atherosclerosis and cardiometabolic disease [7, 20].

In addition to single nutrients, recent large‐scale cohort and multiomics studies propose that dietary patterns and metabolic health are related to coordinated epigenetic signatures affecting cardiovascular aging and risk for disease [128]. These results are consistent with an evolving paradigm from single‐nutrient models to integrated dietary–epigenomic frameworks that may better represent real‐life exposure.

Physical exercise is an especially potent regulator of epigenomic plasticity. Epigenome‐wide studies of ECs, skeletal muscle, and circulating leukocytes show that a subset of the exercise‐induced epigenetic modifications may be paralleled in the cardiovascular tissue. These changes typically induce anti‐inflammatory, antioxidant, and metabolic resilience programs and may partly counteract deleterious epigenetic modifications that are linked to sedentarism and poor metabolic health. Notably, many exercise‐related epigenetic profiles seem to be at least partially reversible, which attests to the strategy of lifestyle interventions that could remold disease‐associated regulatory states rather than simply alleviate downstream pathology [21, 22, 129].

### 3.2 Smoking and Psychosocial Stress

Cigarette smoking is one of the strongest environmental modifiers of cardiovascular epigenetic reprogramming. The smoking‐associated changes in DNA methylation have been consistently found across tissues and populations, with the strongest evidence supporting the role of loci related to inflammation, vascular tone and oxidative stress. Interestingly, some smoking‐associated methylation changes seem to be maintained for years after quitting and suggest an imprint of long‐term epigenetic memory that could influence the remaining vascular risk despite cessation of the exposure [13, 14, 15].

Psychosocial stress is a more diverse yet also emerging exposure that impacts cardiovascular epigenetic regulation. This stress‐related epigenetic remodeling may influence neuroendocrine signaling, immune activation, and endothelial function with secondary potential impact on cardiovascular risk. Crucially, stress‐induced epigenomic remodeling frequently intersects with genetic vulnerability, socioeconomic status, and early adversity, further complicating causal inferences in human studies of such dynamics [4, 23, 130].

### 3.3 Environmental Pollution and Radiation

Exposure to environmental pollutants—including particulate matter, heavy metals, and industrial chemicals—has been associated with increased cardiovascular morbidity and mortality. Epigenetic analyses suggest that such exposures induce widespread alterations in DNA methylation and chromatin accessibility, particularly at genes involved in oxidative stress responses, inflammation, and endothelial dysfunction. These epigenetic changes may sensitize cardiovascular tissues to secondary insults and accelerate disease progression, acting as molecular amplifiers of environmental risk [26, 27, 28].

Radiation exposure, although less prevalent in the general population, offers a paradigmatic example of how environmental stress can induce persistent epigenetic remodeling. Experimental and clinical observations indicate that radiation‐associated epigenetic alterations may contribute to long‐term vascular dysfunction and elevated cardiovascular risk, reinforcing the broader principle that environmental insults can leave durable epigenetic imprints with functional consequences [24].

### 3.4 Integration and Translational Implications

Exposures to the environment and lifestyle factors are not isolated. Rather, additive and interactive effects of exposures over the life course determine the cardiovascular epigenome, leading to heterogeneous patterns of epigenetic change that contribute to explaining interindividual differences in disease susceptibility and progression. This integrative outlook emphasizes that a single‐exposure framework is insufficient and that exposome‐based, longitudinal investigation should be pursued [4, 5, 127].

**FIGURE 6:**
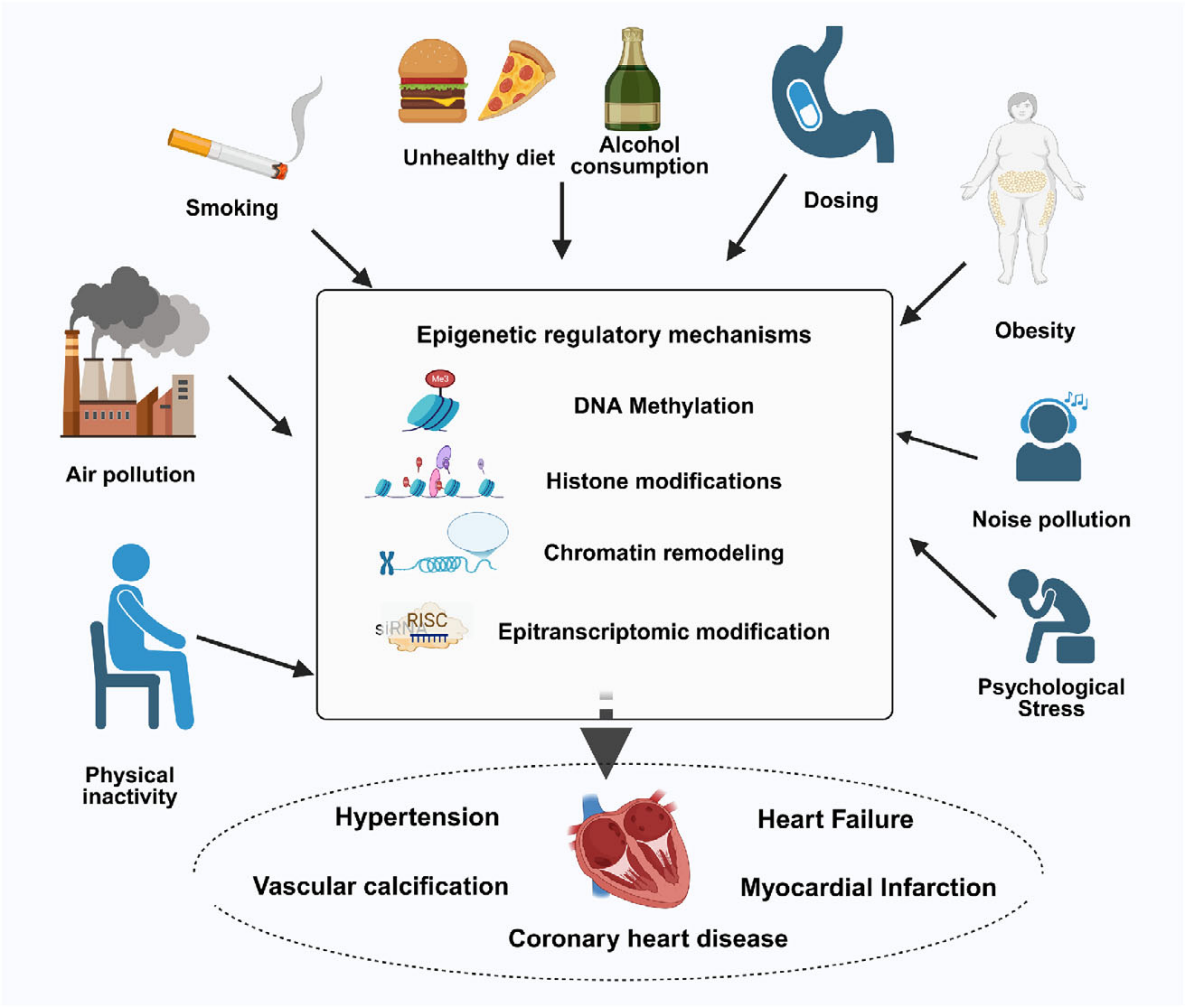
Environmental factors and epigenetic mechanisms in CVDs. Environmental and lifestyle factors—such as smoking, poor diet, air pollution, sedentary behavior, and stress—drive cardiovascular disease by inducing epigenetic modifications. These exposures alter DNA methylation, histone modifications, chromatin remodeling, and RNA modifications, contributing to hypertension (HTN), vascular calcification, CAD, HF, and MI.

Furthermore, the somewhat incomplete reversibility of some epigenetic changes and early onset of maladaptive regulatory states implies that lifestyle interventions (in particular if applicable at an earlier stage) could complement pharmacological approaches in modulating established disease rather than treating it with drugs. A schematic representation showing how environmental and lifestyle exposures impact on epigenetic modulation in cardiovascular cell types during disease stages is given in Figure 6.

## 4 Epigenetic Biomarkers in Cardiovascular Diseases

Epigenetic biomarkers are becoming more and more attractive for cardiovascular risk stratification, diagnosis, and prognosis. Epigenetic marks differ from static genetic variants in that they are able to capture not only the inherited risk, but also the cumulative environmental exposure, and so represent dynamic regulatory states which can change over the course of disease. In CVDs, the most widely proposed epigenetic biomarker candidates are based on DNA methylation profiles and circulating ncRNAs, followed by contributions from chromatin‐based features and multiomics integrative strategies [29, 30, 31].

A fundamental challenge in this area is to differentiate between epigenetic biomarkers that offer incremental and clinically actionable insights compared with those primarily driven by downstream consequences of tissue damage or remodeling. In turn, replication in independent cohorts, consistency across tissues or assay platforms, temporal stability and added value beyond extant clinical risk models are essential criteria for assessing biomarker utility [31, 83]. Without meeting these criteria, epigenetic signals risk remaining descriptive rather than translational.

### 4.1 DNA Methylation‐Based Biomarkers

DNA methylation signatures represent the most mature class of epigenetic biomarkers in cardiovascular research. Large‐scale epigenome‐wide association studies (EWASs) have identified CpG sites and regional methylation patterns associated with atherosclerosis, coronary artery disease, HF, and cardiovascular mortality. Importantly, a subset of these methylation markers has demonstrated reproducibility across independent cohorts and remains associated with outcomes after adjustment for traditional risk factors, supporting their potential clinical relevance [29, 30, 31].

Beyond individual loci, composite methylation scores and epigenetic aging estimators have emerged as integrative biomarkers that capture cumulative biological stress and cardiovascular risk. Accelerated epigenetic aging has been consistently associated with increased incidence of cardiovascular events and adverse outcomes across diverse populations. Although these measures are not disease specific, they provide a quantitative framework for incorporating epigenetic information into cardiovascular risk prediction and may complement—rather than replace—existing clinical scores [32, 33]. Recent studies further suggest that cardiovascular‐specific methylation signatures may outperform generic aging clocks in selected disease contexts, particularly when evaluated longitudinally [131].

However, these developments come with significant restrictions. DNA methylation is strongly tissue‐specific and, at present, most human studies are utilizing peripheral blood as a surrogate for cardiovascular tissues. In addition, differences between the platforms and preprocessing pipelines as well as between analysis strategies further complicate comparability across studies and standardization. These issues highlight the necessity of standardized methodologies, prospective validation and cautious interpretation before routine clinical application [31].

### 4.2 ncRNA Biomarkers

Circulating ncRNAs, especially those related to cardiac epigenetic regulation like miRNAs, have been widely studied as minimally invasive CVD biomarkers. Differential circulating miRNA signatures have been described for MI, HF, and vascular disease that can replicate across cohorts and diseases [34, 35, 36]. Their relative biofluid stability and accessibility through blood‐based assays provide practical advantages for clinical translation.

Nevertheless, the biomarker potential of ncRNAs is constrained by several factors. Many reported associations lack mechanistic specificity and may reflect downstream transcriptional responses rather than upstream regulatory events. Moreover, circulating ncRNA levels are influenced by comorbidities, pharmacological treatments, and technical variables related to sample handling and normalization. These limitations highlight the importance of prioritizing ncRNAs with demonstrated epigenetic functions and robust validation across independent populations, rather than relying on single‐cohort discovery studies [37].

### 4.3 Chromatin‐ and Multiomics‐Derived Biomarkers

Recent advances in epigenomic profiling have enabled exploration of chromatin‐based biomarkers derived from histone modification patterns, chromatin accessibility, and higher order genome organization. Although currently constrained by tissue accessibility and technical complexity, integrative multiomics analyses suggest that chromatin‐level features may provide complementary information to DNA methylation and RNA‐based biomarkers, particularly by capturing cell‐type‐specific regulatory states associated with disease progression [38, 39].

Single‐cell and cell‐free epigenomic approaches further expand the biomarker landscape by enabling resolution of heterogeneous cellular contributions to cardiovascular pathology. While these technologies remain largely confined to research settings, early studies indicate that they may eventually improve specificity for disease‐relevant cell populations and help disentangle causal regulatory changes from secondary effects of tissue remodeling [82, 132, 133].

### 4.4 Translational Considerations and Clinical Integration

For epigenetic biomarkers to achieve meaningful clinical utility, integration with established cardiovascular risk models is essential. Current evidence suggests that epigenetic markers are most effective when used to refine risk stratification or prognosis, rather than as standalone diagnostic tools. Longitudinal studies assessing temporal stability, responsiveness to intervention, and cost‐effectiveness will be critical for defining appropriate clinical use cases and identifying patient populations most likely to benefit from epigenetic risk assessment [31, 83].

A structured summary of validated and emerging epigenetic biomarkers—including molecular type, sample source, associated cardiovascular conditions, and diagnostic or prognostic relevance—is provided in Table 3. Additional candidate biomarkers and supporting studies are systematically summarized therein to ensure comprehensive literature coverage without excessive citation density in the main text.

**TABLE 3:** Screening and identification of epigenetic biomarkers in blood and tissues.

Biomarker name	Molecular type: miRNA/DNA methylation/histone modification, and so forth	Sample source: Plasma/serum/myocardial tissue/PBMCs, and so forth	Detection method: qPCR/MeDIP‐seq/ChIP‐seq, and so forth	Associated disease type: CAD/HF/HTN, and so forth	Clinical relevance (diagnosis/prognosis/subtyping, etc.)	References
lncRNA GAS5	lncRNA	Plasma	RT‐qPCR	CAD	Diagnostic: Positively correlated with stenosis severity; may serve as a biomarker for CAD management	[134, 135]
lncRNA GASL1	lncRNA	Plasma; Myocardial tissue	RT‐qPCR	CHF; HF	Prognosis: Associated with poor prognosis but may improve CHF; Therapeutic: Valsartan improves heart failure via regulation of the PI3K/AKT pathway	[93, 136]
miR‐450b‐5p	miRNA	Myocardial tissue	RT‐qPCR, Western blotting, dual‐luciferase reporter assay	MI	Therapeutic: Serves as a therapeutic target (EGCG exerts antiferroptosis effects through it)	[137]
lncRNA PDE4DIP‐P6	lncRNA	Plasma	RT‐qPCR	MI	Diagnostic: Assists in diagnosing NSTEMI and enhances the diagnostic performance of hs‐cTnT	[138]
lncRNA NEAT1	lncRNA	Myocardial tissue; Plasma	Western blotting, ELISA, immunohistochemistry (IHC) staining, TUNEL staining; RT‐qPCR	Myocardial injury; CAD	Therapeutic: Serves as a potential treatment target to ameliorate myocardial injury; reflects CAD stenosis severity, inflammation, and lipid levels	[139, 140]
lncRNA XIST	lncRNA	Myocardial tissue; Serum	RT‐qPCR; Western blot; ELISA	MI; Myocardial IRI	Therapeutic: Involved in regulating cardiomyocyte apoptosis; protects against myocardial IRI	[65, 141]
miR‐132	miRNA	Peripheral blood; Plasma	RT‐qPCR	HF	Diagnostic: A biomarker downregulated in HF patients; Therapeutic: Serves as a target for HF treatment, as CDR132L can inhibit its activity	[142, 143]
circACAP2	circRNA	Plasma; Myocardial tissue	RT‐qPCR	MI	Diagnostic: Upregulated in the plasma of MI patients; increased expression in cardiomyocytes after MI, promoting apoptosis	[112, 144]
miR‐223	miRNA	Myocardial tissue; Serum	RT‐qPCR	Post‐MI cardiac fibrosis; CAD	Therapeutic: Inhibition of miR‐223 can ameliorate cardiac fibrosis and improve cardiac function; Prognostic: Predicts cardiovascular mortality in CAD patients	[145, 146]
lncRNA TUG1	lncRNA	Serum; Plasma	RT‐qPCR	CHF; MI	Diagnostic: Identifies CHF and evaluates disease severity and prognosis; serves as a diagnostic biomarker for MI	[147, 148]

## 5 Epigenetic Therapeutic Strategies in Cardiovascular Diseases

Considering increasing evidence for the contribution of epigenetic dysregulation in CVD pathogenesis, many epigenetic‐based therapeutic approaches have gained interest. In contrast to classical pharmacological strategies that primarily focus on single signaling cascades, epigenetic interventions seek to reprogram higher order gene‐regulatory programs which are amalgams of multiple disease inputs. This theoretical advantage suggests the potential to impact inflammation, fibrosis, metabolic reprogramming, and cellular plasticity concurrently. On the other hand, the pleiotropic nature of epigenetic control also poses complexity‐ and risk‐related issues if not directly related to specificity, safety and long‐term implications with potentially undesirable effects particularly in the context of chronic CVDs where maladaptive proatherogenic epigenomic states could be established and resistant to intervention [4, 5].

Importantly, recent comprehensive appraisals in HF highlight that reversible epigenetic marks often occur early in disease progression and may signal actionable nodes for therapy, but translating these insights into clinical benefit remains in its infancy. Epigenetic therapies therefore retain substantial promise yet are fundamentally limited by concerns over specificity, delivery, and unintended disruption of basal gene networks. These considerations suggest a model in which epigenetic interventions may be most effective when deployed as adjunctive strategies that augment rather than replace established therapies, particularly in early or moderately advanced disease states characterized by regulatory plasticity [149].

### 5.1 Targeting DNA Methylation and Histone‐Modifying Enzymes

Pharmacological targeting of DNA methyltransferases and histone‐modifying enzymes represents the most extensively investigated epigenetic therapeutic approach in CVD. Preclinical models have shown that inhibitors of DNA methyltransferases or histone deacetylases (HDACs) can attenuate pathological cardiac hypertrophy, fibrosis, and inflammatory activation. These effects are often associated with partial normalization of disease‐associated transcriptional profiles, suggesting modulatory impacts on maladaptive gene programs rather than global epigenomic reversion [84, 86, 87, 88, 150].

Nevertheless, translation to clinical cardiovascular therapy has been limited. Most Despite these promising experimental findings, clinical translation has been limited. Many epigenetic inhibitors currently available were developed for oncological indications and lack cardiovascular cell‐type specificity, raising concerns about off‐target toxicity and deleterious effects on physiological gene regulation. For example, early HDAC inhibitors exhibited broad enzymatic inhibition profiles that compromised homeostatic processes in nontarget cells and have yet to demonstrate clear cardiovascular benefit in controlled clinical studies. Safety concerns are paramount given the chronic nature of CVD management and the need for sustained dosing regimens [85, 131, 151].

Furthermore, recent reviews highlight that the therapeutic window for classical epi‐drugs might be small: indeed it seems to depend on appropriate timing, dosage, and stage of the disease being treated while decreasing with progression or even fixed maladaptive remodeling. These dynamics further emphasize the requirement for next‐generation agents with improved selectivity and delivery strategies, as well as more effective linkage with patient stratification biomarkers to optimize benefit and minimize risk [149].

### 5.2 Targeting Chromatin Readers and Enhancer‐Associated Regulation

Beyond writers and erasers, chromatin “readers”—such as bromodomain and extra‐terminal domain (BET) proteins—have emerged as alternative therapeutic targets. By modulating the interpretation of histone acetylation marks, BET inhibitors aim to selectively suppress stress‐induced transcriptional programs while preserving basal gene activity. One clinically relevant example is apabetalone, a BD2‐selective BET inhibitor that has been evaluated for its effects on inflammation, lipid metabolism, and reverse cholesterol transport in CVD contexts, illustrating how reader modulation can influence disease‐relevant pathways [152, 153].

However, enhancer‐targeted therapies confront unresolved challenges. Disease‐associated enhancers are often highly cell‐type specific, complicating targeted delivery within the heterogeneous milieu of the heart and vasculature. Additionally, redundancy and plasticity within enhancer networks may limit durable therapeutic responses, particularly in advanced disease. These considerations reinforce the need for integrative approaches that combine enhancer targeting with phenotypic stratification and precise temporal modulation of gene expression programs rather than blanket suppression of regulatory elements [154].

### 5.3 RNA‐Based and Programmable Epigenetic Therapies

RNA‐based therapeutic strategies that target epigenetically active ncRNAs or RNA modification pathways represent an emerging frontier in epigenetic therapy. Experimental modulation of selected miRNAs and lncRNAs has demonstrated the capacity to influence chromatin states and transcriptional networks implicated in cardiac remodeling and fibrosis. In particular, early clinical development of ncRNA‐targeted strategies—including antisense oligonucleotides and nanoparticle‐based delivery systems—suggests translational potential, albeit with unresolved issues related to delivery efficiency, specificity, and immunogenicity [48, 94, 155].

Programmable epigenetic editing technologies, such as CRISPR/dCas9‐based approaches, have been proposed as next‐generation tools for locus‐specific modulation of epigenetic marks without global disruption of regulatory machinery. These methods offer conceptual advantages over traditional inhibitors but remain constrained by technical barriers in delivery, off‐target effects, reversibility, and regulatory considerations, limiting their immediate applicability in CVD contexts. As a result, such technologies are currently best categorized as high‐potential future directions rather than ready‐for‐clinic therapies. [136].

At present, these technologies are best viewed as conceptual tools that inform future therapeutic directions rather than near‐term clinical solutions.

### 5.4 Therapeutic Timing, Safety, and Translational Barriers

A major challenge of epigenetic therapy across a number of approaches is the timing of treatment with respect to malignant transformation. Optimally, the most potent clinical benefit of epigenetic therapy is achievable when intervention is implemented at early or intermediate stages of disease in which regulatory plasticity allows widespread alteration of cellular state; unfortunately advanced disease stages frequently exhibit stably entrenched compartments that resist systematic rewiring. Such temporal dependence might, indeed, explain some of the disconnections between strong preclinical efficacy and limited clinical activity seen with certain epigenetic agents [5].

Other translational challenges are the interindividual epigenomic variability, the scarce biomarkers for patient stratification, and lack of clarity about best dosing schedules, as well as the risk of cumulative toxicity with long‐term therapy. As a result, epigenetic treatments are less likely to replace current cardiovascular therapies than work as adjuncts that augment the beneficial effects that artificial and lifestyle interventions impart.

**FIGURE 7:**
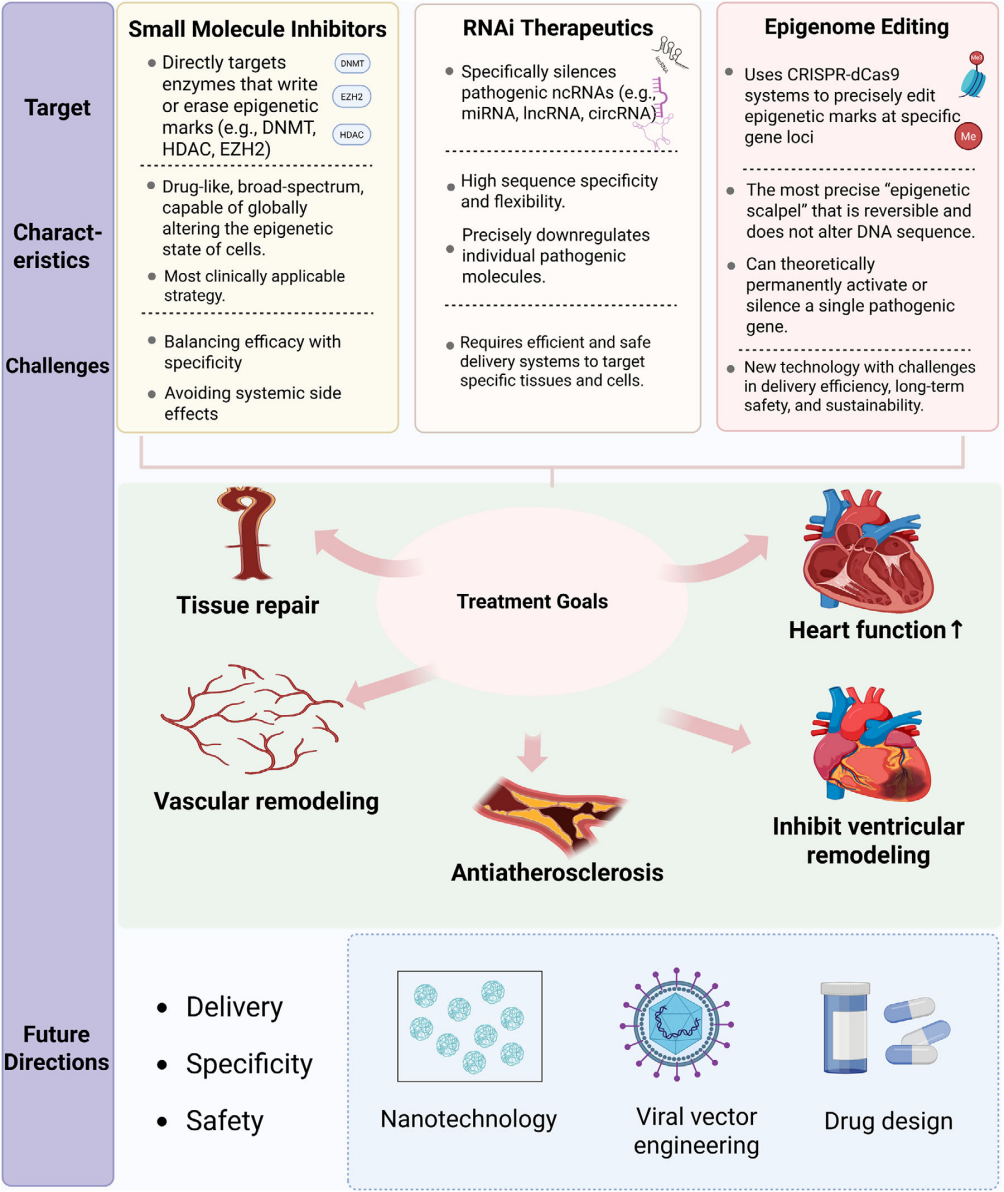
Epigenetic‐targeted therapeutic strategies in CVDs. Three main approaches of epigenetic‐targeted therapies in CVDs are depicted: small molecule inhibitors, RNA interference (RNAi), and epigenetic editing. These therapies include those against epigenetic enzymes (DNMTs, HDACs, and EZH2), modulators of ncRNAs, and the adaptation of CRISPR‐dCas9 technology to cause targeted manipulations of the epigenome. These approaches attenuate pathological remodeling, rescue cardiac function, and advance the goals of personalized precision medicine.

A comparative summary of epigenetic targets for therapy, their mode of action, experimental models and translation status, with emphasis on differences between preclinical and near‐clinical strategies, is shown in Figure 7 and Table 4. Collectively, this evidence illuminates the potential of epigenetic therapies and also the significant hurdles for their translation into cardiometabolic therapy.

**TABLE 4:** Small molecules and their corresponding inhibitors (epigenetic regulation‐targeted therapeutic strategies for CVDs).

Small molecule compound name	Primary target/inhibitor type	Mechanism of action (brief description)	Research stage	References
GSK‐J4	JMJD3/UTX (H3K27me2/3 demethylase inhibitor)	Inhibits H3K27me2/3 demethylases (JMJD3/UTX), enhances H3K27me3 methylation levels, and promotes osteogenic differentiation and calcification of AVICs Inhibits H3K27 demethylases (JMJD3/UTX), restores esophageal smooth muscle–skeletal muscle boundary abnormalities, and alleviates esophageal dilatation Inhibits H3K27me3 demethylases (JMJD3/UTX); monotherapy partially rescues embryonic lethality, while combination therapy ameliorates cardiac valvular enlargement	Cell experiments (AVICs osteogenic induction model) Animal model validation (BRAF <sup>Q241R/+</sup> mice) Animal model (embryonic intervention, BRAF <sup>Q241R/+</sup> mice)	[156, 157, 158]
CPI‐455	KDM5 (H3K4 demethylase inhibitor)	By inhibiting H3K4me3 demethylases (KDM5), increases H3K4me3 modifications on key cardiac developmental genes, thereby promoting differentiation of human pluripotent stem cells into cardiomyocytes By inhibiting H3K4me3 demethylases (KDM5), elevates H3K4me3 modification at the SOX9 promoter, releasing KDM1A‐mediated transcriptional repression of SOX9, thereby promoting cardiomyocyte apoptosis	In vitro stem cell model (human‐induced pluripotent stem cells differentiated into cardiomyocytes) In vitro cell model (HL‐1 cardiomyocyte hypoxia‐reoxygenation injury model)	[159, 160]
IOX1	KDM4 (Jumonji demethylase inhibitor)	By inhibiting the m^6^A RNA demethylase ALKBH5, corrects tricarboxylic acid (TCA) cycle metabolic disorders during myocardial ischemia/reperfusion injury, thereby alleviating myocardial damage	Animal model validation (delivered via targeted nanocages HSSS for treating acute MI in mice)	[161]
Vorinostat	HDAC1/2/3 inhibitor	By inhibiting HDACs, induces hyperacetylation of histones inK562 cells, enhances transcriptional dysregulation, and exerts selective cytotoxicity As a broad‐spectrum HDAC inhibitor, promotes Foxp3^+^ Treg cell differentiation and inhibits PD‐1 signaling, restoring immune homeostasis and significantly attenuating pulmonary vascular pathology in PAH models (more pronounced efficacy in females)	Clinical translation stage (FDA‐approved for T‐cell lymphoma; experimental studies ongoing for CML indications) Preclinical animal model validation (PAH rats and Treg‐deficient mice); not yet tested in clinical trials for PAH indication	[162, 163]
Nicotinamide	SIRT1 activator (NAD^+^ precursor)	Alleviates systemic comorbidities (aging, HTN, metabolic syndrome) and enhances myocardial energy metabolism As an NAD^+^ precursor, increases SIRT1/3 activity → reduces titin acetylation and enhances SERCA2a activity	Preclinical animal model validation (aged mice, hypertensive rats, ZSF1 obese rat models) Animal models plus human observational studies	[164, 165]
Resveratrol	SIRT1/3 activator	Modulates foxo3a/foxo1b expression → alleviates mitochondrial damage → suppresses ROS accumulation → improves calcium dynamics (↓CaD90) → rescues HF phenotype Activates Sirt1 → decreases Smad3 acetylation → inhibits TGF‐β/Smad3 signaling → attenuates myocardial fibrosis, inflammatory infiltration (↓M1 macrophages/↑M2 macrophages), and oxidative stress in HFpEF mice, thereby improving diastolic dysfunction	Preclinical multimodel validation (zebrafish HF model; human AC16 cardiomyocytes) Preclinical animal model validation (mouse HFpEF model induced by unilateral nephrectomy plus aldosterone infusion)	[166, 167]
JQ1 (BRD4 inhibitor)	BET protein (BRD2/3/4) inhibitor	Inhibits BET protein BRD4, thereby blocking profibrotic gene transcription → significantly suppresses cardiac fibroblast activation and collagen secretion, ameliorating myocardial fibrosis in mouse models of pressure overload and MI Selectively inhibits the epigenetic reader function of BRD4, preventing TGF‐β–induced genome‐wide chromatin redistribution of BRD4 (particularly at enhancers/superenhancers) → suppresses RNA polymerase II activation → downregulates profibrotic genes (e.g., Sertad4) → blocks the transition of cardiac fibroblasts to myofibroblasts	Preclinical animal models Preclinical mechanistic studies (rat primary cardiac fibroblasts and mouse pressure‐overload models)	[168, 169]
5‐Aza	DNMT inhibitor	Activates the PI3K/GSK3β‐mtKATP signaling axis → improves mitochondrial function, reduces oxidative stress → attenuates myocardial ischemia/reperfusion injury (↑29% cell survival rate, reduced infarct size) Restores p‐p38 phosphorylation in macrophages → inhibits macrophage‐to‐myofibroblast transition (MMT) → alleviates post‐MI inflammatory dysregulation and cardiac fibrosis in IKKε knockout mice, improving cardiac function Activates the IRE1α‐EGFR‐ERK1/2 signaling cascade, specifically stabilizing the ARE1 element in the 3′‐UTR of LDLR mRNA → markedly increases LDLR protein expression in human hepatocytes, enhancing LDL clearance	Preclinical multimodel validation (H9C2cells, ex vivo rat hearts, in vivo coronary ligation model); preclinical animal studies (IKKε KO mouse MI model); in vitro validation (primary human hepatocytes and Huh7 hepatoma cells)	[170, 171]
RG108	Non‐nucleoside DNMT inhibitor	Inhibits DNMTs, partially preventing TAC‐induced aberrant DNA methylation in cardiomyocytes (↓ ∼2% global methylation), reversing the downregulation of key genes such as Atp2a2(SERCA2a) and Adrb1 (β1‐adrenergic receptor) → attenuates cardiac hypertrophy in rats (↓23% heart weight/body weight ratio), improves contractile function, and reduces fibrosis DNMT inhibition alleviates Atp2a2 (SERCA2a) promoter hypermethylation → partially reverses impaired contractility and systolic/diastolic velocity caused by pressure overload	Preclinical animal model (rat transverse aortic constriction [TAC] model, 4‐week intervention) Effective in vitro (engineered heart tissue [EHT] afterload model)	[172, 173]
Decitabine	DNMT1 inhibitor	Reduces methylation of fetal hemoglobin (HbF) genes in adult erythrocytes → reactivates HbF expression → interferes with sickle hemoglobin polymerization, ameliorating the pathology of sickle cell disease (SCD) Decreases DNA methylation in tumor cells → enhances tumor antigen presentation → synergizes with PD‐1 inhibitor (pembrolizumab) to activate antileukemic T‐cell responses, applied in relapsed/refractory acute myeloid leukemia (R/R‐AML)	Phase II (DECIDER trial, NCT04464772; oral THU–decitabine formulation) Phase I clinical trial	[174, 175]
Antagomir‐21	miR‐21 antagonist	Specifically blocks miR‐21 function → completely abolishes the cardioprotective effects of Na_2_S Silencing miR‐21 → disrupts the STAT3–miR‐21 positive feedback loop → inhibits STAT3 phosphorylation and downstream fibrotic signaling → attenuates atrial electrical and structural remodeling, preventing postoperative AF	Mechanistic research tool (in vitro cell studies and miR‐21 knockout mouse models) Preclinical mechanistic validation (animal models and in vitro studies, not yet applied in clinical therapy)	[176, 177]
2‐APQC	SIRT3 agonist (camptothecin derivative)	Specifically activates SIRT3 → regulates mitochondrial homeostasis (↑PYCR1‐mediated proline metabolism, ↑AMPK–Parkin axis to suppress necrosis) → simultaneously inhibits three profibrotic pathways (mTOR–p70S6K, JNK, TGF‐β–Smad3) → markedly ameliorates isoproterenol (ISO)‐induced cardiac hypertrophy (↓60%) and fibrosis (↓55%)	Preclinical animal models (ISO‐induced rat HF model plus SIRT3 knockout mice to validate target specificity)	[177]
Astragaloside IV	DNMT3a inhibitor	Binds with high affinity and degrades pathological lncRNAs (mouse lnc9456/human lnc4012) → disrupts lncRNA–G3BP2 interaction → inhibits NF‐κB nuclear translocation → reverses cardiomyocyte hypertrophy and cardiac dysfunction (↓ post‐MI ventricular remodeling, ↑ ejection fraction)	Preclinical animal models (mouse MI model plus validation inhuman HF tissues)	[178]
Darutigenol	AKT1 agonist (derived from *Siegesbeckia orientalis*)	Binds with high affinity and activates AKT1 (molecular docking binding energy ≤ ‐9.2 kcal/mol) → suppresses cardiomyocyte apoptosis (↓ caspase‐3 by 45%) and oxidative stress (↓ ROS by 52%) → markedly improves MI and ischemia/reperfusion injury in mice (↓ fibrosis by 40%, ↑ cardiac function by 35%)	Preclinical animal models (mouse MI/reperfusion models); a natural bioactive compound derived from *Siegesbeckia orientalis*	[179]
MC1568	Class IIa HDAC selective inhibitor	Specifically inhibits class II HDACs → disrupts PPARγ/RAR‐mediated differentiation signaling → blocks adipogenesis (in 3T3‐L1 cells) and visceral endoderm differentiation (in F9 cells), with PPRE‐Luc reporter mice confirming suppression of PPARγ signaling in cardiac and adipose tissues Selectively inhibits HDAC4/6 → shows no significant effect on pulmonary vein automaticity or calcium homeostasis (3.1 Hz vs. 2.8 Hz in controls), indicating that class II HDACs do not directly participate in AF‐related electrophysiological remodeling	Preclinical mechanistic studies (cell differentiation models and transgenic mice; nontherapeutic development stage) Mechanistic control tool (nontherapeutic application)	[180, 181]

## 6 Preclinical and Clinical Evidence Supporting Epigenetic Interventions

It has been suggested that epigenetic dysregulation is involved in the entire spectrum of CVD initiation and progression, although the translational maturity of epigenetic interventions reflects a wide gradient among various gene‐regulatory targets and therapeutic approaches. The full potential of the pharmacologic modulation of epigenetic control in cardiovascular therapy has, however, not yet been realized, despite a wealth of mechanistic and preclinical work. This discrepancy mirrors biocomplexity, but also the contrast between epigenetic modulation and standard drug development philosophy, in which sustained reprogramming rather than acute pathway block is the aim.

To provide an overview of the current translational landscape, Table 5 presents landmark preclinical and clinical studies in CVD that leveraged epigenetic pathways, according to epigenetic target, mechanism of action, disease indication, grading of evidence, and status of clinical trials. This systematic review identifies points of concordance—where evidence for experimental efficacy supports clinical feasibility—and enduring gaps that hinder translation.

**TABLE 5:** Representative preclinical and clinical studies targeting epigenetic pathways in cardiovascular diseases.

Epigenetic target	Therapeutic strategy	Disease context	Model/trial type	Trial ID	Phase	Status	Primary objective	Key references
DNA methylation (DNMTs)	Genetic or pharmacological DNMT modulation	Cardiac hypertrophy, fibrosis	Murine pressure overload; cardiac fibroblasts	—	Preclinical	Completed	Assess role of DNA methylation in pathological remodeling	[182, 183]
Histone deacetylases (HDACs)	HDAC inhibition (e.g., Vorinostat/SAHA)	Oncology trials with CV safety monitoring	Human clinical trial	NCT00440115	I/II	Completed	Evaluate safety and tolerability	[184]
BET proteins	BET inhibition (Apabetalone, RVX‐208)	Coronary artery disease	Human clinical trial (ASSERT)	NCT01058018	II	Completed	Reduce vascular inflammation and atherosclerosis	[185]
BET proteins	BET inhibition (Apabetalone)	Type 2 diabetes with recent ACS	Human clinical trial (BETonMACE)	NCT02586155	III	Completed	Reduce major adverse cardiovascular events	[184]
Chromatin remodeling (SWI/SNF)	Genetic modulation of remodeling complexes	Cardiac hypertrophy	Murine genetic models	—	Preclinical	Completed	Define chromatin remodeling in stress adaptation	[186, 187]
Epigenetically active ncRNAs (lncRNAs/miRNAs)	ncRNA perturbation	Fibrosis, inflammation	Murine and cellular models	—	Preclinical	Completed	Regulate chromatin‐associated transcription	[188, 189]
RNA methylation (m^6^A machinery)	METTL3/METTL14 modulation	Cardiac stress responses	Murine and cellular models	—	Preclinical	Completed	Control stress‐responsive gene expression	[190, 191]

Physiologic adaptation and pathologic remodeling are not isolated regulatory layers but rather represent highly interconnected processes.

### 6.1 Evidence From Preclinical Animal and Cellular Models

Among them, preclinical studies in animal and cell models offer the most solid causative relationship evidence for epigenetic implications in CVD. Genetic modification and pharmacological targeting of epigenetic modulators show that changes in DNA methylation, histone modifications, chromatin remodeling, ncRNA action, or RNA methylation can have a direct impact on pathological phenotypes such as hypertrophy, fibrosis, inflammation, endothelial dysfunction, and detrimental vascular remodeling [84, 86, 87, 88].

Crucially, these models show that the effects of epigenetic interventions are not only tissue‐ and cell‐type‐specific, but also highly context‐sensitive. The same regulatory targets can have different results depending on cell types, the stage of the disease, or duration of the intervention and type of pathological stimulus. For instance, superenhancer and chromatin‐associated manipulations often have been more effective in early‐ or mid‐stage disease with substantial epigenomic flexibility compared to late‐stage malignancies, which do not possess regressible regulatory architectures [4, 5].

Preclinical studies also demonstrate that epigenetic regulation is a network‐level phenomenon. Instead of acting via individual downstream targets, epigenetic interventions oversee concerted transcriptional programs that cover such realms as inflammation, metabolism, extracellular matrix handling, and stress‐response pathways. While this network‐level modulation provides a mechanistic explanation for wide‐ranging phenotypic effects, it also raises concerns for off‐target effects and long‐term safety, especially in chronic disease settings.

Beyond proof‐of‐principle studies, a number of genetically or pharmacologically manipulated models offer strong causal evidence for the involvement in cardiovascular pathology of defined epigenetic regulators. For instance, selective suppression of class I and II HDACs decreases pressure overload‐induced hypertrophy and fibrosis in rodents, also accompanied by normalization of stress‐responsive transcriptional programs [172, 192]. Consistently, on‐target DNA methylation modification has been reported to control detrimental remodeling in engineered heart tissue and in vivo hypertrophy models, confirming a regulatory rather than an epiphenomenon role [155, 172].

### 6.2 Human Observational Studies and Epigenetic Signatures

In humans, evidence of association between epigenetic regulation and CVD comes mainly from observational and cohort studies. On an epigenome‐wide basis, DNA methylation signatures, ncRNA profiles, and measures of epigenetic aging have been shown to associate with cardiovascular risk, disease severity, and clinical outcomes. While these results suggest translational applicability, they are by nature correlative and cannot infer causality [29, 33]. Of note, methylation‐ and RNA‐based epigenetic signatures have been shown to be reproducible across cohorts and to retain prognostic value when considered simultaneously with traditional clinical factors, indicating that they are complementary and not merely redundant information content [146, 193].

However, a number of the epigenetic markers have replication across independent cohorts and some maintain their prognostic value after adjustment for traditional cardiovascular risk factors. These observations raise the possibility that epigenetic information adds value to risk stratification and outcome prediction when combined with conventional clinical models. Nevertheless, different tissue sources, analytical pipelines, and cohort composition still hamper generalizability and clinical applicability [31, 83].

Crucially, Table 5 makes no reference to observational epigenetic association studies in a deliberate decision to concentrate on interventional evidence from both preclinical animal models, and clinical trials of interventions targeting such pathways. This contrast is indicative of evidential weight and translational maturity, as well as the synergy that exists between studies using observational versus interventional designs to progress cardiovascular epigenetic research.

### 6.3 Early‐Phase Clinical Trials Targeting Epigenetic Pathways

Very few epigenetic therapies have been tested directly in clinical trials for CVD. The existing clinical evidence is mostly derived from early‐phase testing or from trials designed as primary or secondary preventive trials for non‐CVDs. It is of note that targeting BET proteins with apabetalone is one of the few examples that has progressed to late‐phase clinical testing in a relevant cardiovascular population, offering important lessons on both promise and pitfalls for epigenetic modulation in CVD (Table 5).

Compared to oncology and inflammatory diseases, interventional clinical studies on epigenetic pathways are still rare in the cardiovascular field. The majority of epigenetic agents reviewed to date were not designed/profiled for cardiovascular indications, and the clinical use of these drugs has been limited by concerns over specificity, toxicity, as well as long‐term safety. Not in the first place intended for cardiovascular indications, however, epigenetic modulators are studied in early‐phase clinical trials conducted within noncardiac settings, granting safety and feasibility insights. For instance, low‐dose DNA methyltransferase inhibition and antisense‐based targeting of epigenetically active miRNAs show acceptable tolerability in human subjects, thereby guiding potential repurposing studies in the setting of CVD [143, 194]. Consequently, direct clinical evidence for epigenetic therapies in CVD is lacking [85, 151].

Nevertheless, cardiovascular ramifications of epigenetic modulation are being tested early on in early‐phase clinical and explorative studies either as main interventions or secondary endpoints. Efficacy signals have generally been modest, but these studies also provide valuable data on feasibility, tolerance, and the potential for off‐target effects. Critically, they highlight the importance of development trial designs specific for CVD that include stratification based on disease stage, endpoints informed by mechanism, and patient enrollment guided by biomarkers [195, 196, 197].

### 6.4 Translational Challenges and Future Directions

A number of barriers continue to hinder the transference of epigenetic knowledge into successful cardiovascular therapy. These obstacles are related to limited ability of cell‐type‐selective modulation, lack of certainty when targeting the most relevant therapeutic timing, poor biomarkers for patient stratification, and cumulative toxicities with long‐term administration. Furthermore, CVDs are generally complex, involving multiple factors and having slow progression, which leads to concern about the sustainability and reversibility of epigenetic reprogramming in a long‐term clinical context [4, 5]. Curiously, a number of interventions with strong preclinical efficacy have not led to long‐term clinical benefit, and this has been attributed to the impact of disease chronicity, patient heterogeneity, and compensatory regulatory networks [164].

A summary of preclinical animal studies and human observational analyses, along with novel clinical investigations examining epigenetic pathways in CVD, is reported in Table 5, including modes of action, type of study, models used, and translational phase. Collectively, these data demonstrate both significant gains and barriers that must be overcome before epigenetic therapeutics can become mainstream in cardiovascular medicine.

## 7 Discussion

Epigenetic regulation represents a critical architecture to interpret CVDs as dynamic, context‐dependent processes that are modulated through genetic background and environmental exposure. Emerging data suggest that DNA methylation, histone modifications, chromatin remodeling, ncRNAs, and RNA methylation are coordinated to direct transcriptional programs during cardiovascular development, these processes represent an interactive “epigenetic orchestra” that coordinates metabolic, inflammatory, mechanical, and environmental inputs among various cell types involved in cardiac function.

A key conceptual advance in cardiovascular epigenetics is the recognition of epigenomic plasticity as both an adaptive asset and a potential liability. Cardiovascular cells retain the capacity to dynamically remodel their epigenetic landscapes in response to acute physiological demands and transient stressors. However, chronic or repetitive exposure to pathological stimuli—such as sustained hemodynamic overload, metabolic dysregulation, or persistent inflammation—can progressively stabilize maladaptive epigenetic configurations. Once established, these stabilized regulatory states may perpetuate disease‐associated transcriptional programs independently of the initiating trigger, providing a mechanistic explanation for how transient environmental exposures translate into long‐term cardiovascular risk.

Critically, the dual aspects of epigenomic plasticity provide a fundamental obstacle to causal inference in human studies. EWASs have revealed many DNA methylation and chromatin marks that are associated with cardiovascular phenotypes; however, conventionally designed observational investigations inherently cannot differentiate between epigenetic changes that precede the onset of disease and those induced by the later progression of disease. This uncertainty confounds mechanistic interpretation, and highlights the importance of longitudinal sampling, temporal resolution, and complementary causal inference approaches to establish directionality in the relationship between epigenetic perturbation and disease pathogenesis [198].

This stage‐specific transition from regulatory flexibility to fixation has implications for disease evolution and treatment. Such early plasticity in epigenomic configuration might allow a partial reversal or dampening of maladaptive transcriptional programs. In contrast, advanced stages of disease are frequently predicated by established epigenetic frameworks that prove recalcitrant to reprogramming, which may hinder the effectiveness of late‐stage intervention. This model provides evidence for differences between epigenetic modification patterns at different epochs of disease progression and may unify apparently conflicting results in experimental and clinical studies, as well as underscore the influence of disease stage and cellular context when interpreting epigenetic data.

**FIGURE 8:**
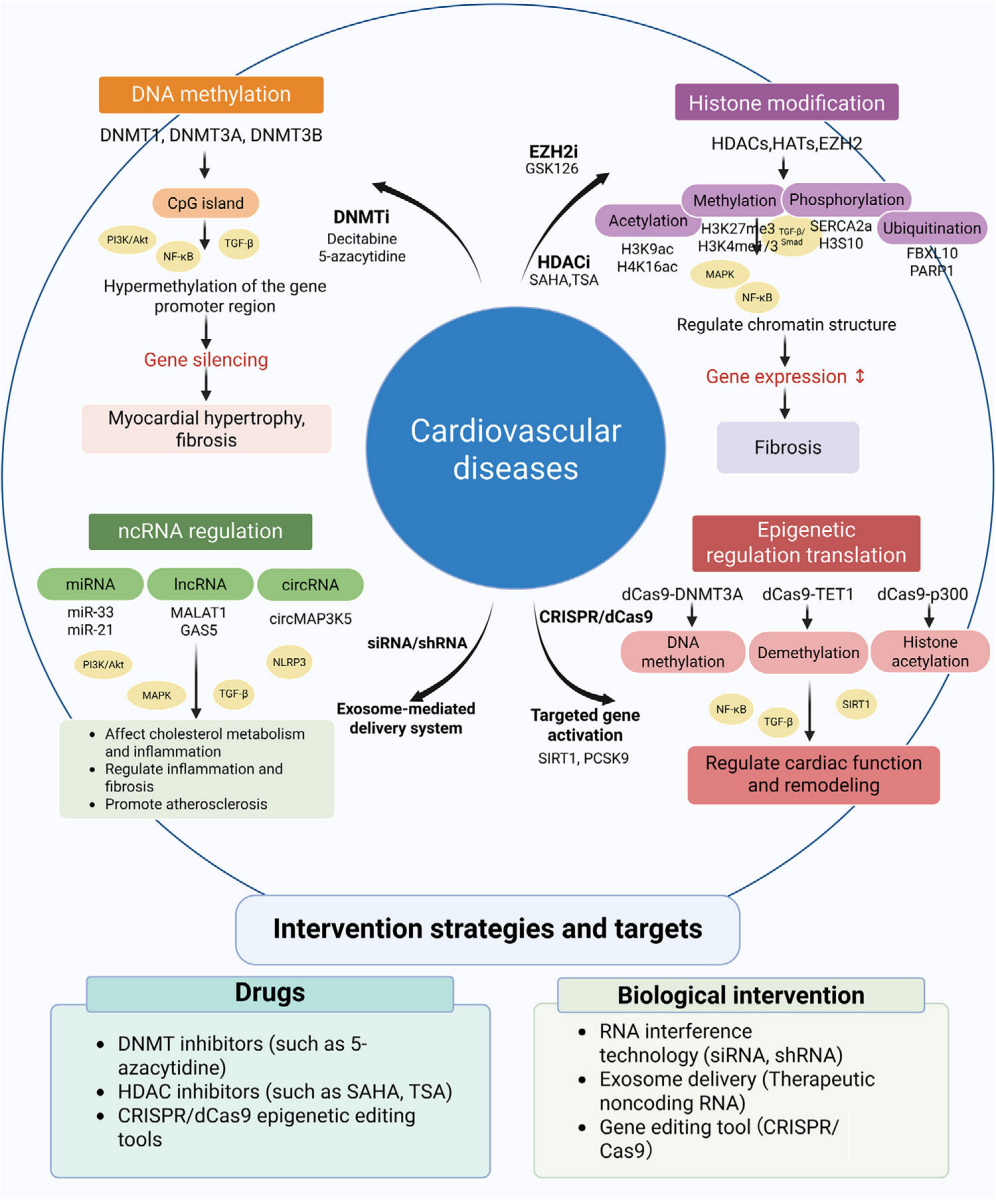
Network of epigenetic regulatory mechanisms and intervention strategies in CVDs. Epigenetic regulation, such as DNA methylation, histone modifications, ncRNAs, and RNA methylation, are crucial in the pathogenesis of CVD. This network combines therapeutic interventions such as DNMT inhibitors (e.g., 5‐Aza), HDAC inhibitors (e.g., SAHA), and CRISPR‐dCas9 genome editing. Pathway‐specific interventions, like the manipulation of SIRT1, PCSK9, and TGF‐β signaling, are also discussed to enhance cardiac repair, modulate gene expression, and slow disease progression.

An epigenetic perspective on environmental exposure, regulatory plasticity, and the trajectory of CVD is schematized in Figure 8. This model suggests that progression of diseases is a matter not only of cumulative molecular damage, but also of gradually stabilizing changes in regulatory networks across multiple epigenetic levels. Concerted changes in enhancer activity, chromatin accessibility, and 3D genome organization reconfigure transcriptional hierarchies in a cell‐type‐specific manner, thereby generating diverse cardiovascular phenotypes. By placing individual epigenetic mechanisms into a common network‐based framework, this model responds to long‐standing divisions in the field and serves as conceptual glue between mechanistic and translational observations.

From the translational point of view, this combined vision comes with a promise and a warning. Epigenetic biomarkers represent a record of cumulative environmental exposure and regulatory state, holding the potential for improved risk stratification and prognostication when added to established clinical models. However, increasing evidence suggests that the reproducibility between epigenetic studies can be affected significantly by differences in analytical pipelines, cohort characteristics, tissue origin and study power and thus is limited for straightforward clinical translation. Without cross‐center validation and standardized methodological standards, epigenetic signatures may get stuck as research tools that never become clinically tractable [199, 200].

Likewise, numerous obstacles related to specificity, delivery, therapeutic timing, and long‐term safety currently hamper the development and clinical implementation of epigenetic therapeutic approaches. The pleiotropic nature of epigenetic remodeling makes it, in principle, equally attractive and uncertain, as it can cause off‐target effects and have multilevel impacts on essential homeostatic gene programs, notably in chronic CVDs. Thus, it appears that epigenetic interventions are unlikely to work as a panacea, acting on a universal disease modifier, and may be useful for a limited number of patient strata and disease stages, still characterized by residual regulatory plasticity.

However, there still exists a number of unsolved issues. Limitations such as the use of bulk‐tissue analyses, restricted availability of cardiovascular tissue from humans, and discrepancies between experimental models and human disease have continued to impede mechanistic dissection. Furthermore, the combined use of single‐cell and spatial epigenomic methodologies has been shown to hold high‐resolution capabilities, which however entail the increased challenges of integrative analyses needed for combined analysis with trajectory inference and scalability for their intended clinical applications.

In the future, we anticipate that further progress in cardiovascular epigenetics will be driven by integrative approaches incorporating single‐cell and spatial epigenomics with longitudinal clinical phenotyping and multiomics integration. Such methods for developing disease‐stage–dependent epigenetic trajectories and windows of regulatory vulnerability could be capable of being targeted. In the end, to gain clinical benefit from our epigenetic knowledge, an evolution from broad modulation toward contextual, network‐informed approaches that acknowledge complexity, adaptivity, and heterogeneity of cardiovascular regulatory systems will be necessary.

## Author Contributions

**Jing‐jing Zhang, Zengwu Wang, and Haoling Zhang**: study concept and design; **Wangzheqi Zhang, Chenyang Mu, Rui Zhao, Runwei Ma, and Xuehai Liu**: drafting of the manuscript. All the authors have read and approved the final manuscript.

## Funding

This research was supported by grants from Science and Technology Department of Yunnan Province—Kunming Medical University, Kunming Medical Joint Special Project—Surface Project, China, No. 202401AY070001‐164; and Yunnan Provincial Department of Science and Technology Science and Technology Plan Project—Major Science and Technology Special Projects, China, No. 202405AJ310003; and the Yunnan Pan Xiangbin Expert Workstation under the Yunnan Provincial Project for Scientific and Technological Talents and Platforms Project, No. 202305AF150069.

## Ethics Statement

The authors have nothing to report.

## Conflicts of Interest

The authors declare no conflicts of interest.

## Data Availability

Data sharing not applicable to this article as no datasets were generated or analyzed during the current study. All information is derived from publicly available articles and datasets.
